# Insulin restores retinal ganglion cell functional connectivity and promotes visual recovery in glaucoma

**DOI:** 10.1126/sciadv.adl5722

**Published:** 2024-08-07

**Authors:** Sana El Hajji, Yukihiro Shiga, Nicolas Belforte, Yves Carpentier Solorio, Olivier Tastet, Philippe D’Onofrio, Florence Dotigny, Alexandre Prat, Nathalie Arbour, Brad Fortune, Adriana Di Polo

**Affiliations:** ^1^ Department of Neuroscience, Université de Montréal, PO box 6128, Station centre-ville, Montreal, Quebec, Canada.; ^2^ Neuroscience Division, Centre de recherche du Centre Hospitalier de l’Université de Montréal (CR-CHUM), 900 Saint Denis Street, Montreal, Quebec, Canada.; ^3^ Discoveries in Sight Research Laboratories, Devers Eye Institute and Legacy Research Institute, Legacy Health, Portland, OR, USA.

## Abstract

Dendrite pathology and synaptic loss result in neural circuit dysfunction, a common feature of neurodegenerative diseases. There is a lack of strategies that target dendritic and synaptic regeneration to promote neurorecovery. We show that daily human recombinant insulin eye drops stimulate retinal ganglion cell (RGC) dendrite and synapse regeneration during ocular hypertension, a risk factor to develop glaucoma. We demonstrate that the ribosomal protein p70S6 kinase (S6K) is essential for insulin-dependent dendritic regrowth. Furthermore, S6K phosphorylation of the stress-activated protein kinase–interacting protein 1 (SIN1), a link between the mammalian target of rapamycin complexes 1 and 2 (mTORC1/2), is required for insulin-induced dendritic regeneration. Using two-photon microscopy live retinal imaging, we show that insulin rescues single-RGC light-evoked calcium (Ca^2+^) dynamics. We further demonstrate that insulin enhances neuronal survival and retina-brain connectivity leading to improved optomotor reflex–elicited behaviors. Our data support that insulin is a compelling pro-regenerative strategy with potential clinical implications for the treatment and management of glaucoma.

## Teaser

Insulin stimulates retinal neuron regeneration, promotes neurorecovery, and restores visual function.

## INTRODUCTION

Individual neurons do not work in isolation and, instead, are part of complex neuronal circuits that establish patterns of synaptic connectivity (*1*). The structural integrity of these neural networks is essential for the optimal function of the central nervous system (CNS) in which dysregulated circuits can initiate a cascade of pathological events that culminate in functional loss (*2*). Preclinical work in rodent models of neurodegeneration provides evidence that circuit alterations result from complex changes in synapses, which disrupt cell excitability and connectivity within local microcircuits, thus promoting subsequent neuronal death (*3*–*8*). Dendritic retraction with synaptic disassembly is one of the earliest changes leading to circuit dysregulation in neurodegenerative diseases (*9*–*15*). Functional deficits often occur before neuronal loss (*16*), thus providing a window of opportunity for the implementation of therapeutic strategies that stimulate dendritic and synaptic regeneration to restore circuit function. CNS neurons, however, have an extremely limited capacity to regenerate, a condition that is only exacerbated by injury or disease (*17*). Therefore, it is critical to identify factors and downstream signaling pathways that influence dendrite regrowth, reestablish functional synapses and connectivity, and enhance neuronal resilience to stress.

Discovered more than 100 years ago (*18*), insulin is a peptide hormone secreted by the pancreas that can cross the blood-brain barrier and activate insulin receptors expressed by neurons and glia (*19*, *20*). In the adult nervous system, insulin exerts a profound influence on physiological processes including energy homeostasis, dendritic plasticity, neurotransmission, circuit function, vascular health, neuronal survival, and cognitive performance (*21*, *22*). Insulin promotes neurite outgrowth by promoting α and β tubulin production, suggesting a crucial role during neurodevelopment (*23*). Insufficient insulin signaling and insulin resistance, observed in prediabetic or diabetic states, have been associated with neurodegenerative diseases (*24*). For example, patients with type 2 diabetes mellitus have an increased risk of developing Alzheimer’s and Parkinson’s disease (*24*–*26*). Insulin dysregulation, independently or concomitantly with hyperglycemia, has been shown to affect key aspects of Alzheimer’s disease neuropathology such as amyloid-β and tau aggregation as well as synaptic loss (*24*). Consistent with this, intranasal insulin administration has been reported to improve memory and mood in these patients (*27*). However, whether insulin promotes dendrite and synapse regeneration leading to circuit function restoration and the mechanisms underlying this response are presently unknown.

To address this knowledge gap, we focused on retinal ganglion cells (RGCs), a population of CNS neurons that serve as the sole output for neurotransmission from the retina to the brain via their axons in the optic nerve. The selective death of RGCs is a central element in the pathophysiology of glaucoma, the leading cause of irreversible blindness worldwide (*28*). There is no cure for glaucoma, and the standard treatment is to reduce intraocular pressure (IOP), a major risk factor for developing the disease. Early retraction of RGC dendrites with synaptic loss is a pathological response in glaucoma, which is highly conserved from mice to humans (*14*, *29*–*32*). Insulin receptors are expressed by adult RGCs (*33*), and deficits in insulin signaling impair neurite outgrowth in these neurons (*34*). Insulin can promote RGC dendrite and synapse regeneration after traumatic optic nerve injury through activation of the mammalian target of rapamycin (mTOR) pathway (*30*). Here, we asked key questions: (i) Can insulin reverse dendritic retraction and synapse loss caused by elevated eye pressure? (ii) Can insulin stimulate circuit reconnection leading to neurorecovery? (iii) What are the molecular effectors involved in insulin-mediated circuit restoration? Our data reveal that insulin is a powerful agent to promote dendrite and synapse regeneration leading to the recovery of light-evoked RGC function and visual behavior. We show that these responses are driven by critical signaling mediators of protein synthesis, metabolism, and growth.

## RESULTS

### Insulin promotes RGC dendrite regeneration during ocular hypertensive stress

**Fig. 1.**
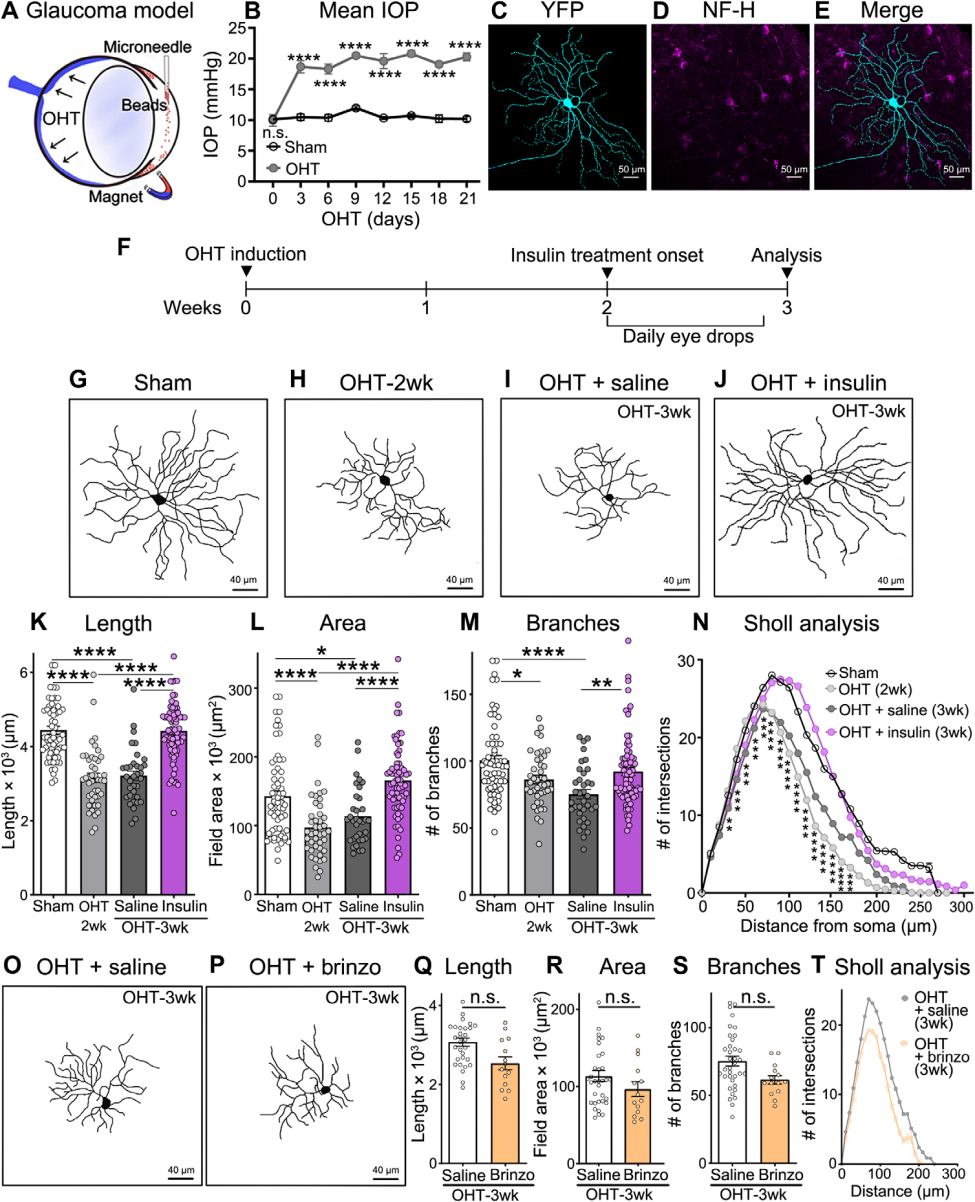
Insulin promotes RGC dendrite regeneration during ocular hypertensive stress. (**A**) Schematic of the mouse glaucoma model induced by intracameral injection of magnetic microbeads, which block aqueous humor outflow leading to ocular hypertension (OHT). (**B**) Intraocular pressure (IOP) increases after microbead injection and remains elevated thereafter [*N* = 5 to 6 mice per group; two-way analysis of variance (ANOVA) with Sidak’s multiple comparisons post hoc test, ****P < 0.0001]. (**C** to **E**) Representative image of an αRGC co-expressing yellow fluorescent protein (YFP) and neurofilament heavy chain protein (NF-H) selected for dendritic arbor imaging and three-dimensional (3D) reconstruction. (**F**) Human recombinant insulin or vehicle (saline) eye drops were administered daily for 1 week starting at 2 weeks after OHT induction, a time when there is already substantial dendrite retraction. Eyes were collected and retinas analyzed 1 week from the onset of insulin treatment (3 weeks of OHT). (**G** and **H**) Representative examples of RGC dendritic arbor skeletons showing dendritic retraction at 2 weeks after OHT induction. (**I** and **J**) Human recombinant insulin, instilled as daily eye drops, promotes robust dendrite regeneration compared to saline-treated eyes quantified at 3 weeks after OHT induction. (**K** to **N**) Quantitative analysis confirmed that insulin restores the process length, arbor area, branch numbers, and complexity (Sholl analysis) (*N* = 5 to 6 mice per group, *n* = 37 to 74 RGCs per group; ANOVA with Tukey’s post hoc test, *P < 0.05, **P < 0.01, and ****P < 0.0001). (**O** and **P**) Representative examples of RGCs from brinzolamide (Brinzo)–treated eyes show marked dendritic retraction. (**Q** to **T**) Quantitative analysis confirmed that lowering IOP alone, in the absence of insulin, is not sufficient to stimulate regenerative growth (*n* = 15 to 41 RGCs per group, *N* = 5 to 6 mice per group; Student’s *t* test). Values are expressed as the means ± SEM. n.s., not significant.

To investigate whether insulin can stimulate dendritic regrowth, we characterized the response of RGCs to ocular hypertension (OHT), a major risk factor to develop glaucoma (*35*). OHT was induced by intracameral injection of magnetic microbeads that, when attracted to the iridocorneal angle with a magnet, blocked aqueous humor outflow and increased IOP (Fig. 1, A and B) (*36*). Microbead occlusion was carried out in mice expressing yellow fluorescent protein (YFP) under control of the Thy1 promoter (Thy1-YFP) (*37*), a transgenic line that allows visualization of individual RGC dendritic trees (Fig. 1C) (*38*). In human retinas, ON and OFF midget RGCs play a fundamental role in vision and account for >80% of all RGCs (*39*). Recent lines of evidence, including integrated single-cell transcriptomics of retinal atlases across species, identified mouse alpha RGCs (αRGCs) as the orthologs of human midget RGCs (*40*). Thus, our analysis focused on αRGCs, which are characterized by strongly labeled somata and large dendritic arbors expressing non-phosphorylated neurofilament heavy chain protein (NF-H) (Fig. 1, C to E) (*41*).

Dendritic arbors were analyzed at 2 weeks after microbead-induced OHT. This time point was selected because high IOP is stable, but no RGC loss is yet detected (fig. S1A) (*36*, *42*, *43*). Figure 1F shows the dosing regimen used in this study: Insulin or vehicle (saline) administration started at 2 weeks after OHT induction and neuronal morphology was analyzed a week later (OHT-3wk). We used human recombinant insulin (Humulin, 100 U/ml: 5-μl eye drop = 0.5 U), a standard commercial formulation used by many patients with diabetes that is readily and widely available. High-resolution images of YFP-labeled RGC dendrites were acquired from retinal flat-mounted preparations using a confocal microscope and three-dimensional (3D) reconstructed to measure dendritic parameters (fig. S1, B to I). Our data show that RGC dendrites undergo significant retraction under OHT stress displaying shorter processes, reduced area, and less branches than sham-operated controls (Fig. 1, G and H). Critically, insulin treatment promoted robust dendritic regeneration (Fig. 1, I and J) and restored the process length, arbor area, and branch numbers, while vehicle-treated eyes showed no improvement (Fig. 1, K to M). Insulin increased the number of branches and promoted a global surge in branch intersections, particularly evident at 50 to 200 μm from the soma, as evidenced by Sholl analysis (Fig. 1N), resulting in enhanced arbor complexity. Overall, insulin restored all dendritic parameters to values similar to those in sham uninjured retinas (sham controls) (Table 1). Daily insulin treatment did not reduce IOP (Table 2); thus, its effect on RGC regeneration could not be attributed to decreasing OHT. To test whether insulin applied topically on the cornea reached the retina and to assess the insulin concentration after treatment, we measured insulin levels in retinal samples using enzyme-linked immunosorbent assay (ELISA). Our data show that retinas collected 30 min after a single insulin eye drop (0.5 U) displayed a sevenfold increase in insulin relative to vehicle instillation (fig. S1J). For example, the mean retinal insulin concentration in eyes treated with a single insulin eye drop was 1.07 ng/ml compared to only 0.15 ng/ml in eyes treated with vehicle. These findings confirm that (i) insulin applied on the mouse cornea reaches the retina; and (ii) exogenous insulin in the retina is within physiological range (nanograms per milliliter), namely, in the same order of magnitude as samples treated with vehicle only (the latter reflects endogenous retinal insulin levels). The dose of topical insulin used here did not reduce blood glucose levels when applied as eye drops in mice (*30*).

**Table 1.** Dendritic parameters after insulin or saline treatment.

Condition	Total dendritic length, μm	Dendritic field area, μm^2^	Dendritic branches, *n*	Animals, *N*	RGCs, *n*
	Means ± SEM	Means ± SEM	Means ± SEM		
**Sham**	4445 **±** 103	142,961 **±** 7375	101 **±** 3 0.7	8	62
**OHT (2 weeks)**	3156 **±** 126	97,081 **±** 6683	86 **±** 3	6	41
**OHT + saline (3 weeks OHT)**	3213 **±** 129	113,501 **±** 7049	75 **±** 3.6	6	36
**OHT + insulin (3 weeks OHT)**	4424 **±** 87	165,667 **±** 5875	92 **±** 3	8	75

**Table 2.** IOP after insulin or saline treatment.

IOP (mm Hg, means ± SEM) after microbead injection								
**Condition**	**3 days**	**6 days**	**9 days**	**12 days**	**15 days**	**18 days**	**21 days**	**Animals, *N***
**OHT + insulin**	18 ± 0.5	18.7 ± 0.5	17.9 ± 0.9	18.7 ± 0.7	18 ± 0.3	18.3 ± 0.5	18.4 ± 0.4	10
**OHT + saline**	17.3 ± 1.6	18 ± 1.05	17.3 ± 0.4	19.3 ± 0.9	17.7 ± 0.3	19.3 ± 0.9	18.2 ± 0.4	7

Insulin-like growth factors are endogenously expressed in the retina (*44*–*47*); hence, we asked whether lowering IOP alone is permissive for RGC dendrite regeneration after retraction. Eye pressure was reduced with brinzolamide, a carbonic anhydrase inhibitor that effectively reduces OHT in microbead occlusion models (*48*) and has negligible effects on neurons or vascular cells (*49*). Brinzolamide treatment started at 2 weeks after microbead injection (eye drops, twice daily: 9 a.m. and 6 p.m.) continuing for 7 days (until 3 weeks of OHT) and effectively reduced IOP to sham-like levels (fig. S1K and Table 3). Our data show that, despite successful eye pressure regulation, there was no dendritic regeneration in brinzolamide-treated eyes relative to eyes treated with saline (Fig. 1, O and P). Quantification of the dendritic length, field area, number of branches, and complexity (Sholl analysis) confirmed that RGCs from eyes that received brinzolamide displayed dendritic pathology to the same extent as saline-treated controls (Fig. 1, Q to T). Collectively, our findings indicate that (i) insulin, provided after RGC dendrites have substantially retracted, promotes notable process regeneration; and (ii) lowering IOP alone, in the absence of exogenous insulin, is not sufficient to stimulate regenerative growth.

**Table 3.** IOP values after brinzolamide or saline treatment.

IOP (mm Hg, means ± SEM) after microbead injection								
**Condition**	**3 days**	**6 days**	**9 days**	**12 days**	**15 days**	**18 days**	**21 days**	**Animals, *N***
**OHT + saline**	17.3 ± 1.6	18.0 ± 1.05	17.3 ± 0.4	19.3 ± 0.9	17.7 ± 0.3	19.3 ± 0.97	18.2 ± 0.4	7
**OHT + brinzo**	16.9 ± 0.47	16.7 ± 1.00	18 ± 0.88	18.4 ± 0.5	11.0 ± 0.83	11.0 ± 0.83	12.26 ± 0.7	9

### Insulin restores excitatory synaptic inputs to vulnerable neurons

**Fig. 2.**
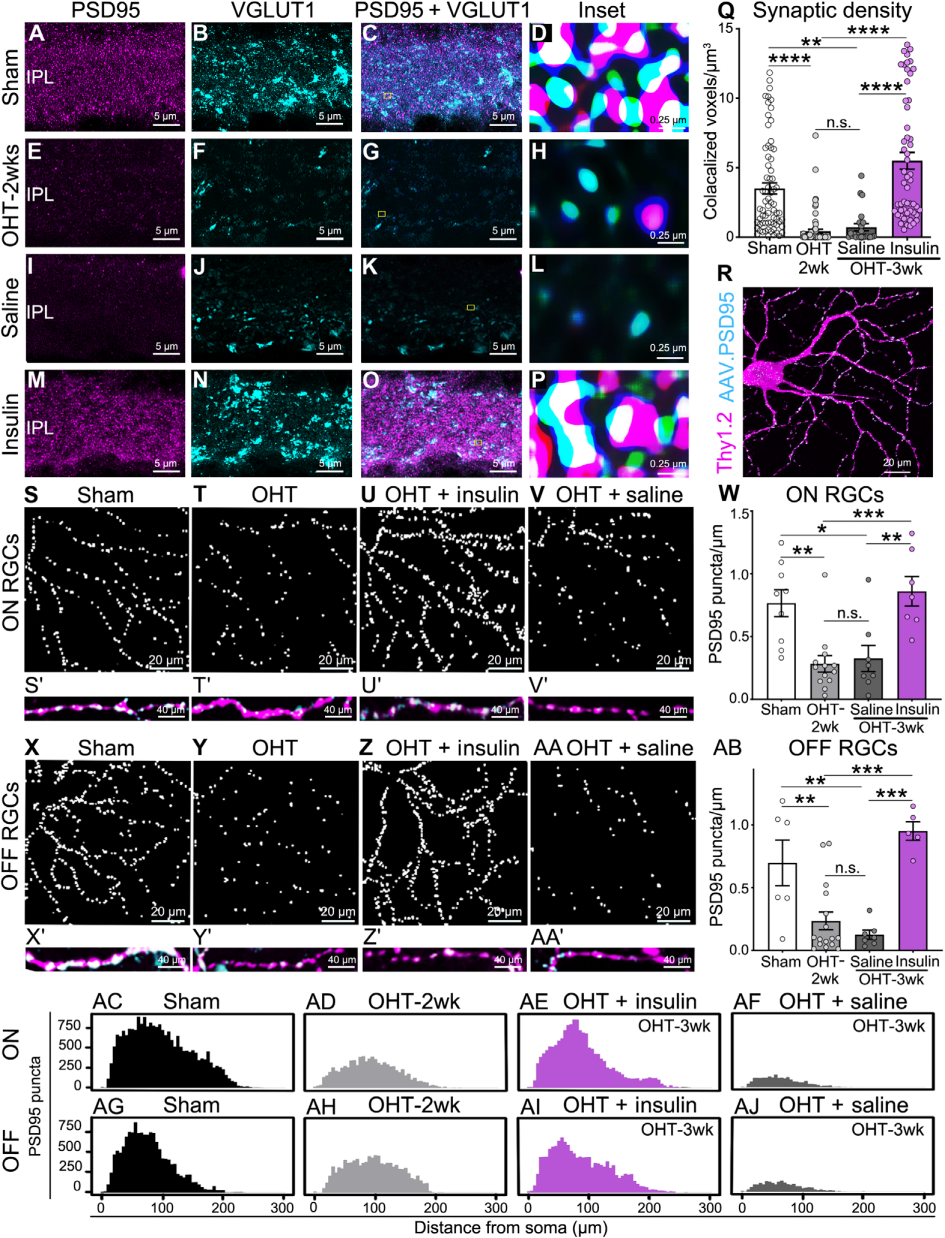
Insulin restores excitatory synaptic inputs to vulnerable neurons. (**A** to **H**) Retinal immunohistochemistry staining of endogenous PSD95 and VGLUT1 shows loss of synaptic components at 2 weeks after OHT induction. (**I** to **P**) Insulin administered after synapse disassembly rescues PSD95 and VGLUT1 expression in the inner plexiform layer (IPL) relative to saline-treated controls. [(D), (H), (L), (P), and (**Q**)] Quantification of pre- and postsynaptic voxels confirmed that insulin promotes robust regeneration of partnered synaptic components compared to vehicle-treated retinas at 3 weeks after OHT induction (*N* = 5 to 6 mice per group; ANOVA with Tukey’s post hoc test, **P < 0.01 and ****P < 0.0001). (**R**) Adeno-associated virus (AAV)–mediated expression of PSD95 in RGCs allows visualization of excitatory synaptic complexes on individual dendritic branches. A significant decrease in the density of excitatory postsynaptic sites was observed in both ON (**S** and **T**) and OFF (**X** and **Y**) RGCs. (**U**, **V**, **Z**, and **AA**) Daily insulin eye drops fully restored the density of PSD95 puncta in both ON and OFF RGC dendrites. (**S′**, **T′**, **U′**, **V′**, **X′**, **Y′**, **Z′**, and **AA′**) High-magnification PSD95 puncta show synaptic density reduction with OHT and regeneration with insulin in both ON and OFF RGCs. (**W** and **AB**) Quantitative analysis confirmed that insulin mediates robust synaptic regeneration to levels similar to sham-operated controls (ON: *N* = 5 to 6 mice per group, *n* = 9 to 13 RGCs per group; ANOVA, *P < 0.05, **P < 0.01, and ***P < 0.001; OFF: *N* = 5 to 6 mice per group, *n* = 7 to 16 RGCs per group; ANOVA, **P < 0.01 and ***P < 0.001). (**AC** to **AJ**) Sholl analysis of synaptic density shows the distribution of PSD95-labeled synapses on dendritic processes, from the soma to the terminal, during glaucoma and after insulin treatment. OHT promoted loss of excitatory synapses along the entire length of the dendrite, both proximal and distal to the cell body and insulin restored synaptic contacts across the full field in both ON and OFF αRGCs. Values are expressed as the means ± SEM.

Excitatory contacts between RGCs and bipolar cells occur at the level of the inner plexiform layer in specialized structures called ribbon synapses (*50*). To investigate whether insulin treatment restored excitatory synapses on regenerating dendritic processes, we first co-localized and quantified changes in endogenous postsynaptic density protein 95 (PSD95), present in RGC dendrites, and vesicular glutamate transporter 1 (VGLUT1), a presynaptic protein expressed in bipolar ribbon synapses (*51*). Previous work demonstrated that presynaptic ribbons in bipolar cell axon terminals and postsynaptic complexes in αRGCs are lost after axonal injury including glaucomatous damage (*30*, *52*–*54*). Consistent with this, we observed a pronounced decrease in immunolabeling of both PSD95 and VGLUT1 at 2 weeks of OHT induction relative to sham controls (Fig. 2, A to H). In contrast, insulin administered after synapse disassembly, as per the regimen used for dendritic analysis shown in Fig. 1F, promoted marked rescue of PSD95 and VGLUT1 expression in the inner plexiform layer relative to saline-treated controls (Fig. 2, I to P). To determine whether synaptic partners, defined as the colocalization of postsynaptic PSD95 and presynaptic VGLUT1 puncta, regenerate with insulin, we performed quantification of pre- and postsynaptic voxels to measure the 3D volume occupied by PSD95 and VGLUT1 in the inner plexiform layer (Fig. 2, D, H, L, and P). Quantitative analysis confirmed that insulin promoted robust regeneration of pre- and postsynaptic partnered synaptic components compared to vehicle-treated retinas (Fig. 2Q).

Next, we sought to understand the distribution of regenerating excitatory synapses in individual RGC dendrites and the response of different neuronal subtypes. For this purpose, we used a serotype 2 adeno-associated virus (AAV) encoding PSD95 fused to a red fluorescent protein (RFP) tag (AAV.PSD95) under the control of the synapsin promoter, which directs selective expression in RGCs (*55*). Thy1-YFP mice received a single intravitreal injection of AAV.PSD95 2 weeks before induction of OHT followed by treatment with insulin or vehicle as above. AAV-mediated PSD95 was robustly expressed in RGCs and allowed clear visualization of excitatory synaptic complexes on individual dendritic branches (Fig. 2R). As with the analysis of dendritic arbors, we focused on αRGCs, identified by their NF-H expression in combination with RFP to visualize AAV-encoded PSD95 (fig. S2). The number of PSD95-positive synaptic puncta that colocalized with YFP-labeled dendrites was quantified and normalized relative to the length of each dendritic process. RGC subtypes were identified on the basis of their dendritic stratification in the inner sublamina (ON) or outer sublamina (OFF) within the inner plexiform layer as described (*30*, *56*). Our data show a significant decrease in the density of RGC excitatory postsynaptic sites relative to sham controls in both ON (Fig. 2, S and T) and OFF (Fig. 2, X and Y) RGCs, with more pronounced synapse loss in OFF neurons (Fig. 2, W and AB), consistent with previous reports (*52*–*54*, *56*, *57*). Notably, daily insulin eye drops fully restored the density of PSD95 puncta in both ON and OFF RGC dendrites (Fig. 2, U to W, Z, AA, and AB). We then analyzed the distribution of PSD95-labeled synapses on dendritic processes, from the soma to the terminal, during glaucoma and after insulin treatment. OHT resulted in loss of excitatory synapses along the entire length of the dendrite, both proximal and distal to the cell body (Fig. 2, AC, AD, AG, and AH), and insulin restored synaptic contacts across the full field in both ON and OFF RGCs (Fig. 2, AE, AF, AI, and AJ). We conclude that insulin restores the density of postsynaptic sites on regenerating RGC dendrites independently of RGC subtype and distance from soma.

### S6K is essential for insulin-mediated RGC dendrite regeneration

**Fig. 3.**
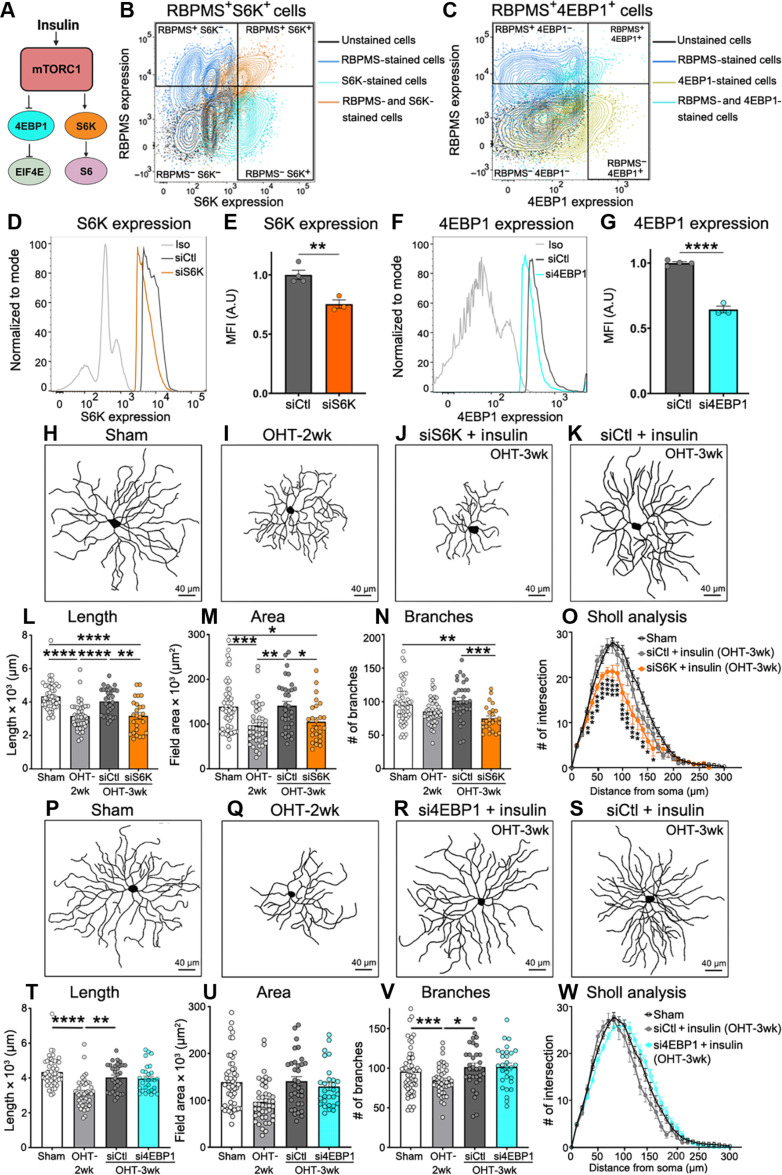
S6K, but not 4EBP1, is essential for insulin-mediated RGC dendrite regeneration. (**A**) Schematic of insulin signaling components with a focus on the mTORC1 downstream signaling molecules S6K and 4EBP1. (**B** and **C**) Flow cytometry contour plot showing the gating strategy based on the fluorescence-minus-one (FMO) controls (RBPMS^−^S6K^−^-, RBPMS^+^S6K^−^-, and RBPMS^−^S6K^+^-stained retinas) to select the RBPMS^+^S6K^+^ cells for analysis of the mean fluorescence intensity (MFI) (B). A similar approach was used to select 4EBP1^+^ RGCs (C). (**D**) Flow cytometry histogram showing the level of S6K protein in RGCs (RBPMS^+^S6K^+^ cells) treated with siS6K or control siCtl. RGCs from retinas that received siS6K show reduced expression of S6K protein relative to siCtl-treated controls. An isotype (Iso) non-targeting antibody is included as control. (**E**) Quantitative analysis of the MFI confirms that siS6K reduces the expression of S6K in RGCs compared to siCtl (*N* = 3 to 4 mice per group, *n* = 30,000 to 50,000 RGCs per retina; Student’s *t* test, **P < 0.01). (**F**) Representative flow cytometry histogram showing the level of 4EBP1 protein in RGCs exposed to si4EBP1 or siCtl. An Iso non-targeting antibody is included as control. (**G**) Quantitative analysis of MFI shows that si4EBP1 significantly reduces the expression of 4EBP1 in RGCs compared to siCtl (*N* = 3 to 4 mice per group, *n* = 30,000 to 50,000 RGCs per retina; Student’s *t* test, ****P < 0.0001). A.U., arbitrary units. (**H** to **K**) Representative examples of RGC dendrites showing process retraction at 2 weeks of OHT [(H) and (I)]. S6K knockdown abrogates insulin-mediated dendrite regeneration [(J) and (K)]. (**L** to **O**) Quantitative analysis confirmed that siS6K leads to a marked reduction in the total dendritic length, arbor area, and number of branches indicative of impaired neuronal complexity (*N* = 5 to 6 mice per group, *n* = 32 to 63 RGCs per group; ANOVA, *P < 0.05, **P < 0.01, ***P < 0.001, and ****P < 0.0001). (**P** to **W**) 4EBP1 knockdown did not alter the pro-regenerative effect of insulin (*N* = 5 to 6 mice per group, *n* = 29 to 74 RGCs per group; ANOVA, *P < 0.05, **P < 0.01, ***P < 0.001, and ****P < 0.0001). Values are expressed as the means ± SEM.

Insulin binding to its receptor stimulates phosphoinositide-3′ kinase and its target Akt, leading to activation of the mTOR complexes 1 and 2 (mTORC1 and mTORC2) (*58*). mTORC1 is primarily involved in cap-dependent mRNA translation and protein synthesis, while mTORC2 regulates the organization of the cytoskeleton and cell growth/proliferation (*59*, *60*). Previous work suggested that mTORC1 mediates the formation of new dendritic branches in injured RGCs, while mTORC2 drives process extension induced by insulin (*30*). However, the role of downstream mTOR effectors in these responses and their contribution to insulin-induced dendrite regeneration are unknown. We first examined the role of mTORC1 targets based on its critical role as a regulator of RGC dendritic arbor morphology (*42*). mTORC1 activates the p70S6 kinase (S6K), leading to phosphorylation of the ribosomal protein S6, while it inactivates the eukaryotic translation initiation factor 4E (eIF4E)–binding protein 1 (4EBP1), a repressor of protein translation (Fig. 3A) (*61*). We used a loss of function strategy by administration of targeted short interfering RNA (siRNA) pools against S6K or 4EBP1 (siS6K and si4EBP1, respectively) concomitant with the onset of insulin treatment (fig. S3A). A non-targeting siRNA was used as control (siCtl). siRNAs were administered by intravitreal injection because this approach results in rapid and preferential uptake by RGCs likely due to the proximity of the ganglion cell layer to the vitreous chamber (*38*, *42*, *62*–*64*). To validate the efficacy of these siRNAs, retinas were collected after siRNA injection, dissociated, fixed, permeabilized, and labeled with antibodies against the RGC-specific marker RBPMS and S6K or 4EBP1, and protein expression was quantified by flow cytometry (Fig. 3, B and C, and fig. S3, B to E). This approach allowed us to assess RGC-specific changes in protein levels, a major advantage over other strategies that report global gene or protein changes from all retinal cells (e.g., quantitative polymerase chain reaction and Western blots). Injection of siS6K or si4EBP1 resulted in a substantial reduction of RGC-specific S6K or 4EBP1 protein expression, respectively, compared to a non-targeting control siRNA (siCtl) (Fig. 3, D to G), but did not change the percentage of cells expressing S6K or 4EBP1 in the total RGC population (fig. S3, F and G).

Our data show that S6K knockdown completely abrogated insulin-mediated dendrite regeneration, resulting in RGCs characterized by rudimentary and shriveled dendritic arbors with shorter and considerably less processes (Fig. 3, H to J). In contrast, eyes treated with insulin and siCtl (control) showed robust regeneration, validating the effect of siS6K (Fig. 3K). Quantitative analysis confirmed that siS6K led to a marked reduction in the total dendritic length, arbor area, and number of branches indicative of impaired neuronal complexity (Fig. 3, L to O). In contrast, 4EBP1 knockdown with si4EBP1 did not alter the pro-regenerative effect of insulin, and RGC dendrites were able to regrow long processes that resulted in large and complex arbors (Fig. 3, P to S). Quantification of dendritic parameters confirmed that reducing 4EBP1 levels did not affect regenerative outcome (Fig. 3, T to W).

**Fig. 4.**
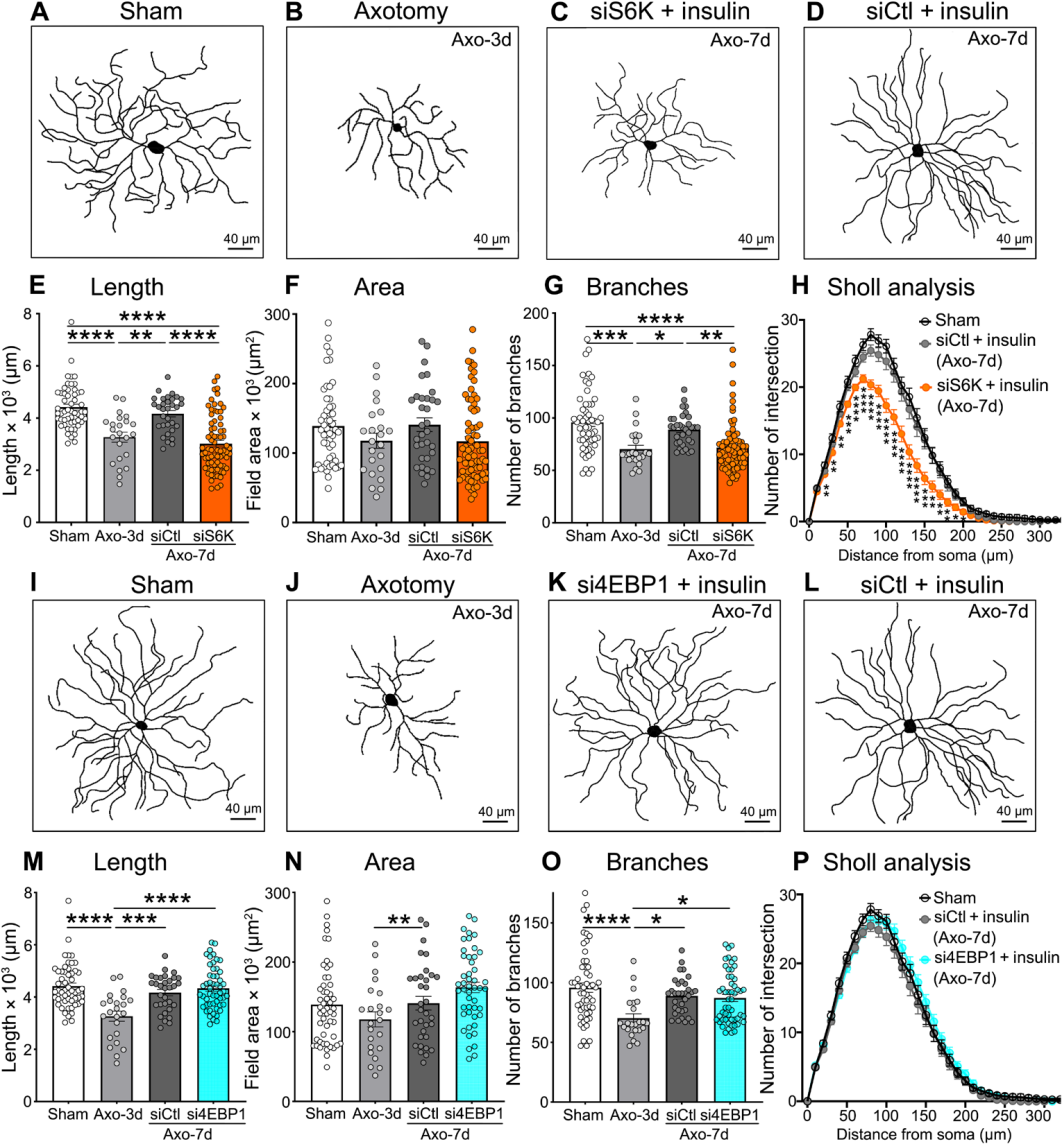
S6K is required for RGC dendrite regeneration after optic nerve axotomy. (**A** to **D**) Representative examples of RGC dendrites showing dendritic shrinkage at 3 days of optic nerve axotomy [(A) and (B)]. Insulin daily eye drops in the presence of siS6K inhibited the pro-regenerative effect of insulin (C) relative to a control siRNA (siCtl) (D). (**E** to **H**) Quantitative analysis shows that siS6K administration blocks insulin-mediated regeneration during axotomy-induced damage (*N* = 5 to 6 mice per group, *n* = 63 to 75 RGCs per group; ANOVA, *P < 0.05, **P < 0.01, ***P < 0.001, and ****P < 0.0001). Values are expressed as the means ± SEM. (**I** to **L**) Representative examples of RGC dendrites showing that 4EBP1 knockdown had no effect on insulin-induced regeneration after optic nerve axotomy relative to a siCtl [(K) and (L)]. (**M** to **P**) Quantitative analysis confirmed that si4EBP1 administration does not block the regenerative effect of insulin after axotomy (*N* = 5 to 6 mice per group, *n* = 54 to 63 RGCs per group; ANOVA, *P < 0.05, **P < 0.01, ***P < 0.001, and ****P < 0.0001). Values are expressed as the means ± SEM.

To determine whether the role of S6K in insulin-mediated dendrite regeneration was specific to OHT damage, we also examined the effect of S6K and 4EBP1 knockdown in a model of traumatic optic nerve injury (axotomy), which triggers rapid and stereotypical RGC retraction preceding neuronal loss (Fig. 4) (*30*, *38*). In this model, marked dendritic retraction is observed at 3 days after injury (*30*); thus, we initiated daily insulin eye drops at this time point and analyzed the dendritic length, area, and complexity 4 days later (7 days after lesion). Our data show that, similar to OHT, S6K silencing impaired insulin-induced dendrite regeneration (Fig. 4, A to H), whereas 4EBP1 knockdown had no effect on dendritic morphology after optic nerve axotomy (Fig. 4, I to P). These data demonstrate that S6K, but not 4EBP1, is essential for insulin-dependent RGC dendrite regeneration and that its role is conserved across modalities of optic nerve injury.

### SIN1-dependent cross-talk between mTORC1 and mTORC2 is required for insulin-driven regeneration

**Fig. 5.**
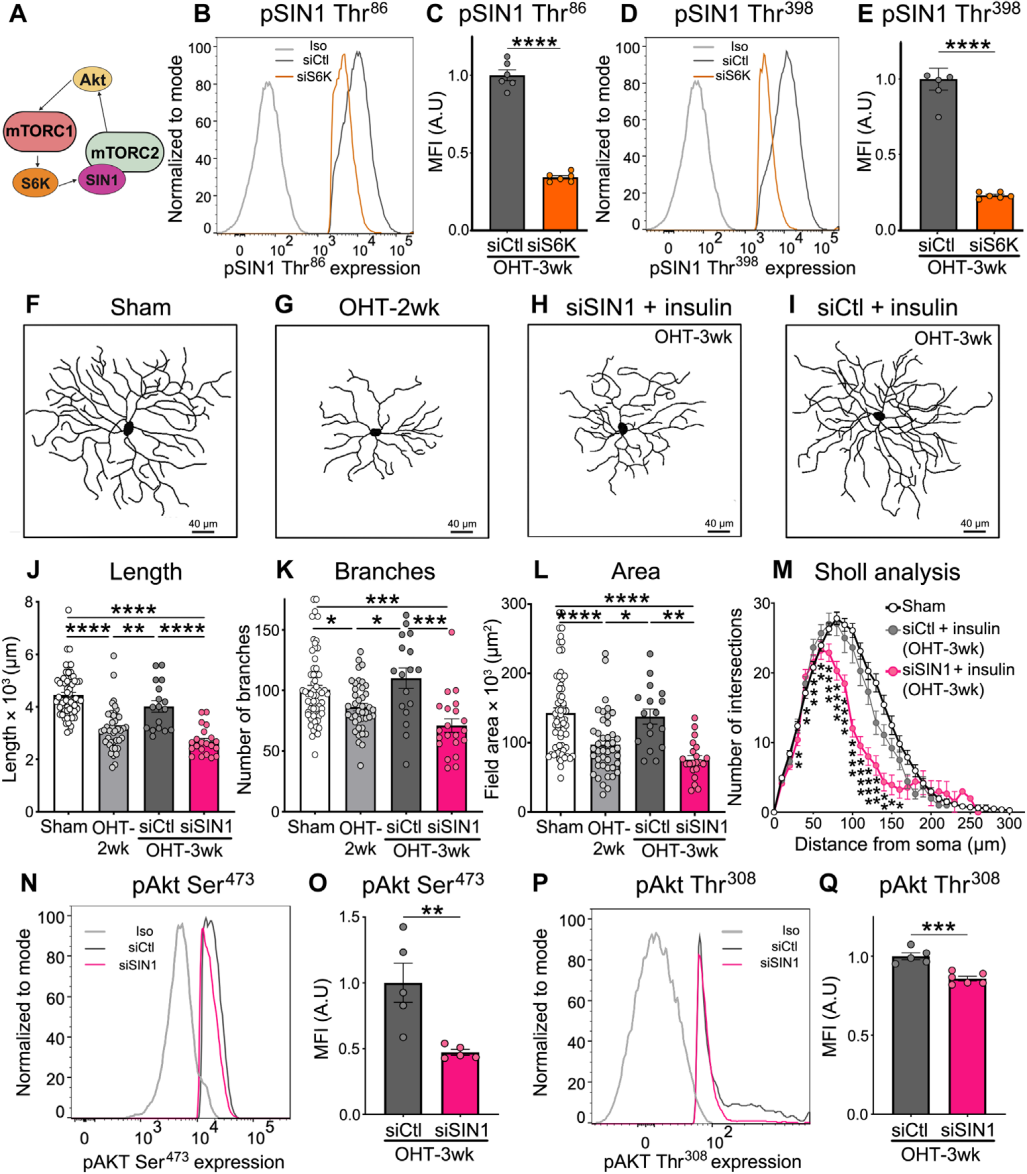
SIN1-dependent cross-talk between mTORC1 and mTORC2 is required for insulin-driven regeneration. (**A**) Schematic diagram of a cross-talk model between mTORC1 and mTORC2. (**B**) Flow cytometry histogram showing the level of phosphorylated SIN1 (pSIN1) at Thr^86^ in RGCs (RBPMS^+^S6K^+^ cells) following siS6K or siCtl administration combined with insulin eye drops during OHT. siS6K effectively reduces the levels of pSIN1 Thr^86^ in RGCs. An Iso non-targeting antibody is included as control. (**C**) Quantitative analysis confirms reduced pSIN1 phosphorylation at Thr^86^ after siS6K treatments relative to siCtl (*N* = 6 mice per group, *n* = 30,000 to 50,000 RGCs per retina; Student’s *t* test, ****P < 0.0001). (**D**) Flow cytometry histogram shows that pSIN1 phosphorylation at Thr^398^ is also reduced in RGCs after S6K knockdown. An Iso non-targeting antibody is included as control. (**E**) Quantitative analysis confirms reduced pSIN1 Thr^398^ phosphorylation with siS6K (*N* = 5 to 6 mice per group, *n* = 30,000 to 50,000 RGCs per retina; Student’s *t* test, ****P < 0.0001). (**F** and **G**) Representative examples of RGC dendrites from eyes subjected to OHT (2 weeks) and sham controls. (**H** and **I**) Eyes with OHT that received siSIN1 or siCtl after insulin treatment show that SIN1 inhibition (H) obliterated the pro-regenerative effect of insulin compared to a siCtl (I). (**J** to **M**) Quantitative analysis confirmed that SIN1 knockdown inhibits the ability of insulin to restore the length, number of branches, field area, and complexity (Sholl analysis) of RGC dendrites (*N* = 5 to 6 mice per group, *n* = 26 to 63 RGCs per group; ANOVA, *P < 0.05, **P < 0.01, ***P < 0.001, and ****P < 0.0001). (**N** to **Q**) Quantitative analysis of flow cytometry showed that siSIN1 administration combined with insulin treatment during OHT significantly reduces the phosphorylation of Akt at Ser^473^ and Thr^308^ (*N* = 5 mice per group, *n* = 30,000 to 50,000 RGCs per retina, Student’s *t* test, **P < 0.01, ***P < 0.001). Values are expressed as the means ± SEM.

Previous studies showed that strategies that block mTORC2 function cause a marked reduction in the dendritic length (*30*, *65*), suggesting that mTORC2 may play a role in dendrite regeneration. Given our finding that S6K is sufficient to promote insulin-mediated dendritic regrowth and complexity (Figs. 3 and 4), we hypothesized that S6K might influence mTORC2 activity. To explore this possibility, we focused on the stress-activated protein kinase–interacting protein 1 (SIN1), an important component of mTORC2 that maintains complex integrity and regulates substrate specificity, hence indispensable for mTORC2 function (Fig. 5A) (*66*). S6K has been shown to phosphorylate SIN1 at two sites: threonine-86 and threonine-398 (Thr^86^ and Thr^398^, respectively) (*67*). However, the manner in which SIN1 phosphorylation by S6K affects its activity has been controversial due to its cell and condition dependency (*68*). Therefore, we first explored the ability of S6K to phosphorylate SIN1 in RGCs and asked whether S6K knockdown affected SIN1 activation during insulin-mediated dendrite regeneration. For this purpose, we administered siS6K or a non-targeting siCtl 2 weeks after OHT induction, concomitant with the onset of insulin eye drops, and assessed SIN1 phosphorylation a week later. SIN1 phosphorylation at Thr^86^ and Thr^398^ selectively in RGCs was quantified by flow cytometry of phosphorylated proteins. Our results show that, when S6K was selectively inhibited, there was a significant reduction in SIN1 phosphorylation at both Thr^86^ and Thr^398^ sites relative to controls treated with siCtl (Fig. 5, B to E) as well as the number of RGCs phosphorylated at these sites (fig. S4, A and B), indicating that SIN1 is a phosphorylation target of S6K in RGCs.

Next, we investigated whether SIN1 is required for insulin-mediated RGC dendrite regeneration using an siRNA against SIN1 (siSIN1) in conjunction with insulin eye drops as shown in fig. S3A. Validation of SIN1 levels after siSIN1 intravitreal injection confirmed that siSIN1 effectively reduced SIN1 expression in RGCs (fig. S4, C to F). Reconstruction and analysis of RGC dendrites from eyes that received insulin in combination with siSIN1 showed severe dendritic retraction and loss of complexity relative to eyes treated with siCtl (Fig. 5, F to I). Quantification of dendritic parameters confirmed that, in the absence of SIN1, insulin cannot promote RGC dendrite regeneration (Fig. 5, J to M). SIN1 is involved in the recruitment of substrates for phosphorylation by mTORC2, notably Akt, which, in turn, can activate mTORC1 (*69*) in a feedforward loop (Fig. 5A). For full activity, Akt needs to be phosphorylated at serine-473 (Ser^473^) and Thr^308^ residues (*67*). To examine whether Akt is a target of SIN1, we administered siSIN1 at 2 weeks of OHT and quantified Akt phosphorylation (pAkt) at Ser^473^ and Thr^308^ after insulin treatment using flow cytometry as above. SIN1 knockdown significantly reduced pAkt at both Ser^473^ and Thr^308^ residues in RGCs (Fig. 5, N to Q) without affecting the number of RGCs expressing these proteins (fig. S4, G and H).

**Fig. 6.**
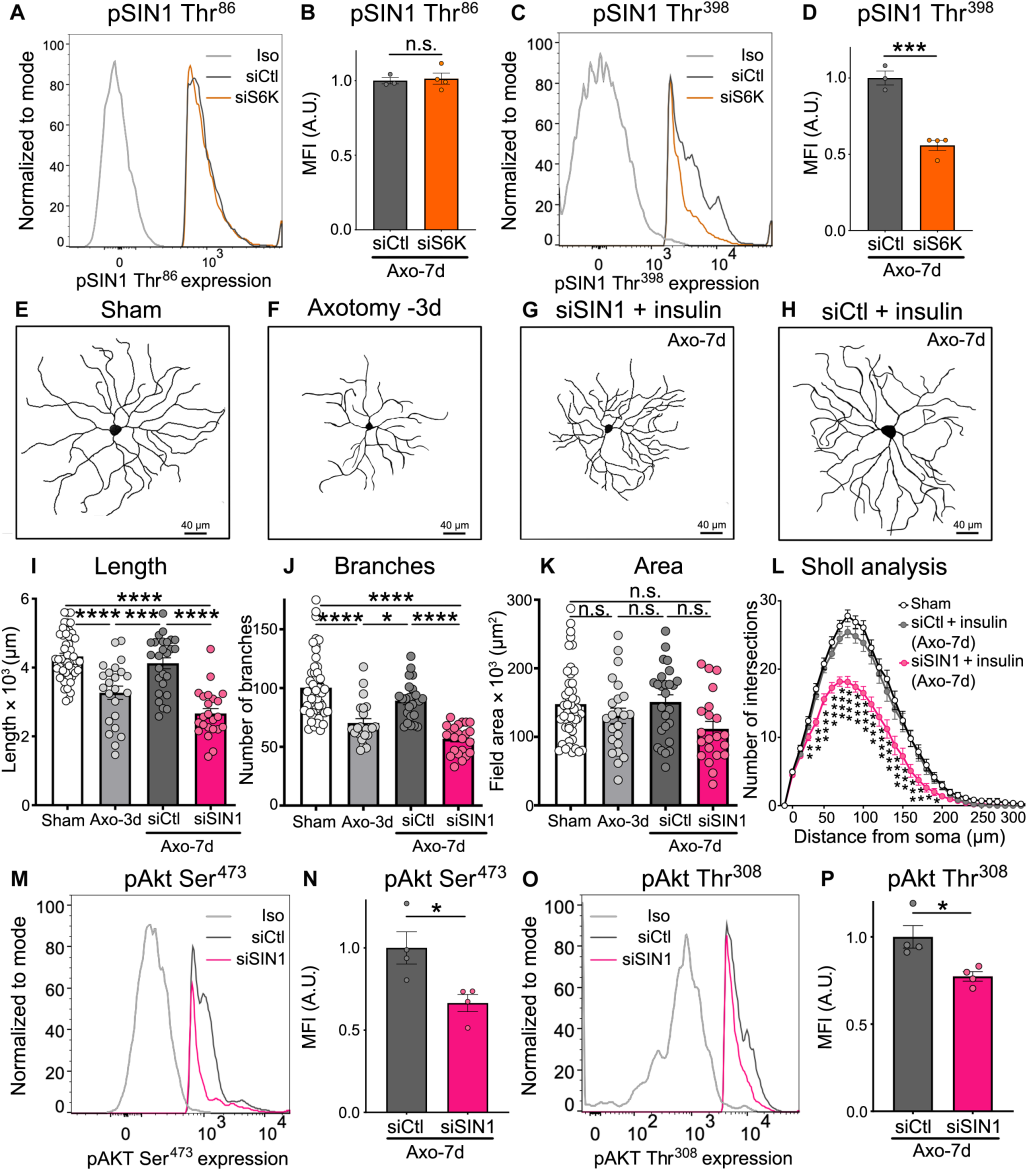
SIN1 mediates insulin-induced RGC dendrite regeneration after optic nerve axotomy. (**A** and **B**) Flow cytometry histogram (A) and quantitative analysis (B) show that siS6K treatment does not reduce RGC-specific SIN1 phosphorylation (pSIN1) at Thr^86^ after axotomy (*N* = 3 to 4 mice per group, *n* = 30,000 to 50,000 RGCs per retina; Student’s *t* test, n.s.). An Iso non-targeting antibody is included as control. (**C** and **D**) In contrast, siS6K substantially reduces the level of pSIN1 Thr^398^ in RGCs compared to siCtl treated retinas (*N* = 3 to 4 mice per group, *n* = 30,000 to 50,000 RGCs per retina; Student’s *t* test, ***P < 0.001). An Iso non-targeting antibody is included as control. (**E** to **H**) Representative examples of RGC dendrites from eyes treated as follows: (i) sham-operated (E), (ii) optic nerve axotomy (3 days after lesion) (F), (iii) axotomy treated with siSIN1 and insulin (7 days after lesion) (G), and (iv) axotomy treated with siCtl and insulin (7 days after lesion) (H). These data show that siSIN1 inhibits the regenerative effect of insulin in axotomized retinas. (**I** to **L**) Quantitative analysis confirmed that SIN1 knockdown inhibits the ability of insulin to regenerate the dendritic length, number of branches, field area, and complexity (Sholl analysis) (*N* = 5 to 6 mice per group, *n* = 26 to 63 RGCs per group; ANOVA, *P < 0.05, **P < 0.01, ***P < 0.001, and ****P < 0.0001). (**M** to **P**) Quantitative analysis of flow cytometry data showed that siSIN1 administration combined with insulin treatment after axotomy significantly reduces the phosphorylation of Akt at Ser^473^ and Thr^308^ (*N* = 4 mice per group, *n* = 30,000 to 50,000 RGCs per retina; Student’s *t* test, *P < 0.05). Values are expressed as the means ± SEM.

In the axotomy model, siS6K significantly decreased SIN1 phosphorylation in RGCs at Thr^398^ but not at Thr^86^ (Fig. 6, A to D, and fig. S5, A and B). Despite this, SIN1 knockdown inhibited insulin-mediated dendritic regrowth (Fig. 6, E to L), suggesting that SIN1 phosphorylation at Thr^398^ is critical for RGC dendrite regeneration. In addition, similar to OHT, SIN1 was required for pAkt at Ser^473^ and Thr^308^ after axotomy (Fig. 6, M to P, and fig. S5, C and D). Moreover, SIN1 knockdown decreased RGC-specific levels of phosphorylated ribosomal protein S6 at Ser^240/244^ residues (pS6) (fig. S5E), a well-characterized and robust target of mTORC1 essential for protein translation (*61*), providing further evidence that mTORC1 activation is SIN1-dependent and supporting a feedforward model. Collectively, these data demonstrate that (i) SIN1 is a target of S6K in RGCs; (ii) SIN1 phosphorylation at Thr^398^ is required for insulin-mediated RGC dendrite regeneration downstream of S6K; and (iii) SIN1 can phosphorylate Akt, which, in turn, further activates mTORC1 leading to S6 phosphorylation, supporting a positive feedback loop. Our data indicate that SIN1 is a key molecular link between mTORC1 and mTORC2 during insulin-induced dendritic regrowth. On the basis of our observation that key signaling components of mTORC1 and mTORC2 feed into each other to activate a pro-regenerative response, we conclude that both mTORC1 and mTORC2 are essential to mediate dendrite regrowth downstream of insulin.

### Insulin stimulates neuronal survival and restores visual function

**Fig. 7.**
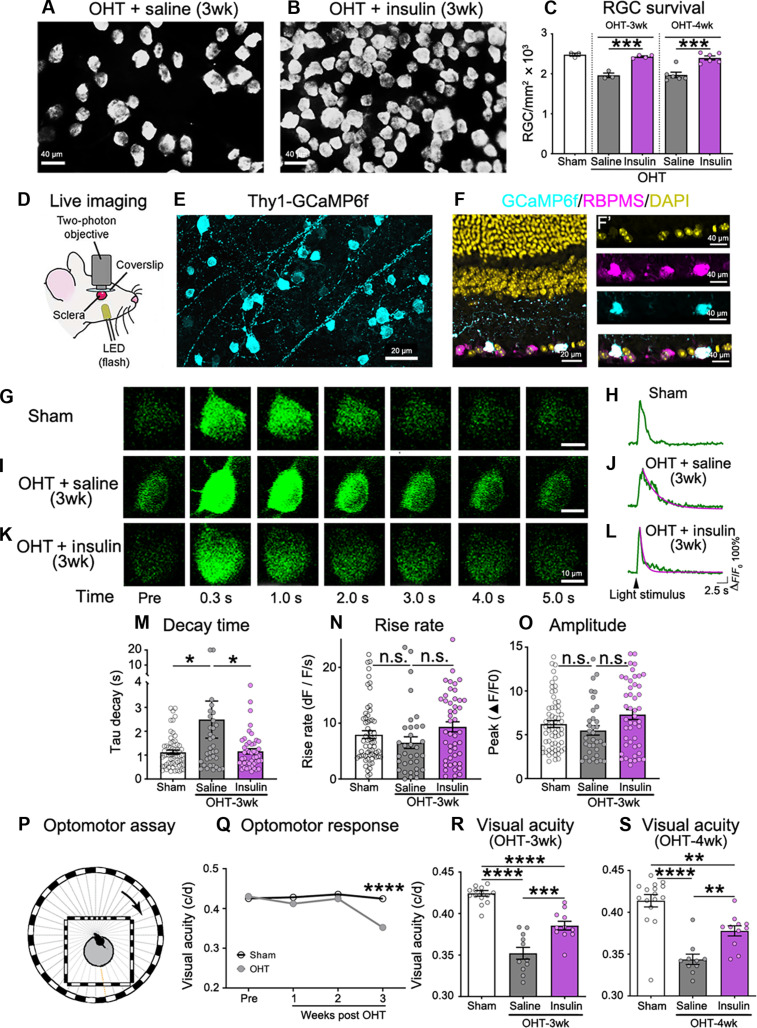
Insulin stimulates neuronal survival and restores visual function. (**A** and **B**) Retinas from glaucomatous eyes treated with insulin or saline labeled with RBPMS at 3 weeks after OHT induction. (**C**) Quantitative analysis shows that insulin promotes RGC survival to levels found in uninjured sham-operated control eyes at 3 and 4 weeks after OHT induction relative to saline controls (*N* = 3 to 6 mice per group; ANOVA, ***P < 0.001). (**D**) Two-photon laser scanning microscopy (TPLSM) setup for live imaging of Ca^2+^ dynamics. LED, light-emitting diode. (**E**) Thy1-GCaMP6f mouse retina showing GCaMP6f-positive RGCs. (**F**) GCaMP6f expression in RGCs is confirmed using RBPMS and 4′,6-diamidino-2-phenylindole (DAPI). (**G** and **H**) Time-lapse images from longitudinal TPLSM recordings of αON-RGC soma, before (Pre) and after light stimulation in sham retinas (G). Light stimulation elicits a brief Ca^2+^ transient with fast signal decay (H). (**I** and **J**) αON-RGCs from OHT eyes treated with saline display increased Ca^2+^signal decay time (J). Exponential fits show the decay time constant (τ). (**K** and **L**) Insulin treatment restores light-evoked Ca^2+^ transient dynamics. (**M** to **O**) Quantitative analysis shows that Ca^2+^ decay time is substantially delayed in glaucoma [(M) longer decay time, gray bar], and insulin improves this response [(M) purple bar]. Ca^2+^ rise rate and peak amplitude do not change across conditions [(N) and (O)] (*N* = 5 to 6 mice per group, *n* = 34 to 61 RGCs per group; one-way ANOVA with Tukey’s multiple comparisons post hoc test, *P < 0.05 and n.s.). (**P**) Optomotor reflex assay setup. (**Q** to **S**) Longitudinal optomotor reflexes before (Pre) and after microbead injection shows progressive visual acuity decay with OHT exposure resulting in significant vision loss by 3 weeks of OHT exposure (*N* = 11 to 14 mice per group; ANOVA, **P < 0.01, ***P < 0.001, and ****P < 0.0001) (Q). Quantitative analysis of the optomotor response at 3 weeks after OHT induction shows a significant improvement in visual acuity in mice treated with insulin relative to saline (*N* = 11 to 14 mice per group; ANOVA, **P < 0.01, ***P < 0.001, and ****P < 0.0001) [(R) and (S)]. Values are expressed as the means ± SEM. c/d, cycles/degree.

Insulin is a potent neurotrophic factor that, concomitantly or as a result of process growth, can stimulate cell survival (*70*). Thus, we examined whether insulin-mediated dendritic regeneration had an impact on RGC viability. Insulin treatment followed the same regimen used for all the experiments in this study (i.e., initiated after 2 weeks of OHT) (Fig. 1F), and RGC density was quantified at 3 and 4 weeks of OHT, a time when there is significant RGC loss in the magnetic microbead model, thus allowing the assessment of neuroprotection (fig. S1A) (*36*). Insulin promoted RGC survival and, notably, supported neuronal density to levels found in uninjured sham-operated control eyes at 3 and 4 weeks after OHT induction, whereas pronounced neuronal death was observed in control saline-treated retinas (Fig. 7, A to C). To evaluate whether insulin enhanced neuronal function, we recorded single-RGC calcium (Ca^2+^) responses in transgenic mice carrying the Ca^2+^ indicator GCaMP6f driven by the Thy1 promoter (Thy1-GCaMP6f). Light-evoked Ca^2+^ signals were visualized using two-photon laser scanning microscopy (TPLSM) (Fig. 7D), allowing minimally invasive trans-scleral imaging with single-cell resolution in living mice (*43*, *55*, *71*). RGC-specific expression of GCaMP6f was confirmed in retinal flat-mounts and cross sections (Fig. 7, E and F). Consistent with our structural analysis, we focused on αRGCs, specifically the ON subtype, a major cell class characterized by maintained firing during the bright stimulus phase (*72*). The identity of ON αRGCs was confirmed by post hoc analysis of dendritic stratification in the ON sublamina as well as high levels of NF-H (fig. S6) (*52*, *72*, *73*). Mice were presented with a light stimulus, and longitudinal real-time videos of Ca^2+^ transients in RGC soma were acquired by TPLSM (Fig. 7, G to L). In sham uninjured retinas, light elicited a burst of Ca^2+^ increase in RGC soma followed by a rapid signal decay (Fig. 7, G and H). In control OHT eyes injected with saline, Ca^2+^ responses were slower and presented a marked delay in Ca^2+^ decay (Fig. 7, I and J). Insulin treatment restored light-evoked Ca^2+^ transient dynamics to values similar to uninjured sham eyes (Fig. 7, K and L). Quantification of Ca^2+^ transient parameters confirmed that insulin eye drops rescued Ca^2+^ dynamics in RGCs, notably shortening the decay time (Fig. 7M). The rise rate and peak amplitude of the responses did not change across groups (Fig. 7, N and O).

To further probe the role of insulin on functional recovery and assess retina-brain connectivity, we measured the optomotor reflex response, a visual behavior characterized by direction-selective head movements used for image stabilization triggered by RGC inputs (*74*). Mice were subjected to longitudinal evaluation of the stereotyped behavior evoked by grating light stimuli (Fig. 7P), and visual acuity was calculated. Visual acuity decreased in eyes with high IOP in a time-dependent manner becoming significantly lower than sham controls at 3 weeks after OHT induction (Fig. 7Q); thus, we measured optomotor responses at 3 and 4 weeks of elevated eye pressure. Insulin eye drops were instilled daily for 1 or 2 weeks, depending on the duration of the experiment (3 or 4 weeks of OHT, respectively). Insulin treatment markedly improved visual acuity in mice with glaucoma relative to saline-treated controls, and this effect was sustained between 3 and 4 weeks of OHT (Fig. 7, R and S). We conclude that daily insulin administration translates into substantial improvements in neuronal function, survival, and circuit connectivity.

## DISCUSSION

Dendritic pathology with synapse disassembly is a common feature of neurodegenerative diseases including glaucoma (*9*–*14*). The identification of strategies that halt, delay, or reverse synaptic failure is crucial to counter neurodegeneration and prevent functional loss. We show that OHT stress, a major risk factor to develop glaucoma, promotes arbor retraction accompanied by a profound loss of excitatory synapses along the entire dendritic field. Topical insulin administration, after dendrite retraction and synapse decay has already occurred, promoted robust process regrowth and restoration of arbor area, complexity, and synaptic connections. Although the relationship between glaucoma and diabetes mellitus has been the subject of debate in the past, cumulative epidemiologic evidence suggests that the risk of developing glaucoma increases in the diabetic population (*75*). Given the potent effect of insulin, it is conceivable that systemic insulin treatment may reduce the incidence of glaucoma in patients with diabetes; however, robust and well-controlled studies showing this association are lacking. In addition, some reports have established a correlation between glaucoma and type 2 diabetes, characterized by the inability of cells to effectively respond to insulin, also known as insulin resistance (*76*–*78*). This is consistent with the association of genetic polymorphisms related to pancreatic β cell function in type 2 diabetes and increased risk of primary open-angle glaucoma (*79*). It is possible that RGCs become unresponsive to insulin in patients with type 2 diabetes, and, although it remains to be tested, this can potentially increase the susceptibility of RGCs to glaucomatous damage.

Our study reports several findings including that (i) insulin promotes RGC dendrite and synaptic regeneration in an inducible mouse model of experimental glaucoma; (ii) S6K, but not 4EBP1, is essential for insulin-dependent RGC dendrite regeneration, a role that is conserved in two paradigms of optic nerve injury; (iii) SIN1 is required for insulin-mediated RGC dendrite regeneration downstream of S6K; (iv) SIN1 serves as a molecular nexus between mTORC1 and mTORC2 to support insulin-induced dendritic regrowth; (v) mTORC1 and mTORC2 feed into each other to activate a positive feedback loop that promotes a robust pro-regenerative response; (vi) insulin promotes RGC survival leading to recovery of light-evoked Ca^2+^ responses during glaucomatous damage; and (vii) insulin treatment improves optomotor responses in mice with glaucoma, a sign of recovery of retina-brain connectivity. Here, we focused on classic αRGCs expressing NF-H, which are characterized by large soma and expansive dendritic fields with a typical branching pattern (*80*), reducing the risk of confounding retraction or lack of regeneration in neurons with more compact dendritic trees. Previous studies reported that RGCs with dendritic stratification in the OFF sublamina are more vulnerable to synaptic alterations in mouse glaucoma models (*52*, *53*, *81*). Consistent with this, we show that OFF αRGCs lose considerably more synaptic contacts during IOP elevation than ON αRGCs. Insulin treatment restored excitatory postsynaptic sites on both ON and OFF αRGC dendrites to densities similar to those found in uninjured sham controls. Sensory neurons in *Caenorhabditis elegans* and *Drosophila* display a robust ability to regrow the stereotypical pattern of dendritic branches after laser-induced dendrite ablation (*82*–*84*), suggesting that spontaneous dendritic regrowth may occur after injury. Insulin-like neurotrophic factors are known to be produced by retinal cells (*44*–*46*) and could promote dendrite regeneration when the source of stress is attenuated. However, our data show that pharmacological reduction of OHT by itself did not restore dendritic arborization, suggesting that endogenous insulin or insulin-related proteins are not sufficient to promote RGC dendritic regrowth and that exogenous insulin administration is essential to trigger this response. Previous studies showed that reducing IOP by itself leads to transient recovery of visual function (*85*, *86*), a response that was also observed following nicotinamide supplementation (*87*). Given the prominent role of insulin on glucose, lipid, and protein metabolism, its action may be through mechanisms similar to those proposed for transient visual function improvement including dendritic and synaptic regeneration (this study) as well as increased mitochondrial function and axonal transport. Along these lines, recent work demonstrated that strategies that restore mitochondrial function and transport along RGC axons also promote visual recovery (*55*).

The powerful pro-regenerative effect of insulin prompted us to investigate its molecular effectors, notably those downstream of the mTOR pathway. mTOR acts as a central hub for multiple signals to control metabolism, cell growth, proliferation, differentiation, autophagy, and apoptosis through the activation of gene transcription and protein synthesis (*88*). Previous studies demonstrated that mTOR activity declines in injured RGCs and that mTOR loss triggers dendrite pathology (*30*, *38*, *42*). For example, RGCs subjected to OHT display early signs of bioenergetic decline characterized by hyperactivation of the energy biosensor adenosine monophosphate kinase (AMPK), reduced adenosine 5′-triphosphate production, and loss of mTORC1 function (*42*, *55*). AMPK hyperactivity inhibits mTORC1 triggering dendrite retraction, synaptic loss, and RGC dysfunction (*42*). On the basis of this, we investigated whether components of mTORC1 signaling, specifically S6K and 4EBP1, were required for insulin-mediated dendrite regeneration. We found that S6K, but not 4EBP1, was essential to unleash the full regenerative potential of insulin. The marked effect of S6K knockdown on the ability of insulin to stimulate dendritic regrowth was observed in both OHT damage and traumatic optic nerve lesion (axotomy), suggesting that this is a conserved signaling mechanism across injury modalities. siRNA-mediated partial knockdown of S6K (~30%) was sufficient to promote a robust effect on dendritic morphology. This finding strongly suggests a key role of S6K within the mTOR signaling complex; therefore, a small change in its function is expected to have a large impact on dendritic structure. This is consistent with previous studies showing that siRNA-mediated attenuation of the expression of key retinal proteins (~20 to 50% reduction) was sufficient to have a potent and sustained biological effect on RGCs (*38*, *42*, *62*–*64*, *89*–*91*). While 4E-BP1 restricts cap-dependent translation in the absence of growth factors and nutrients, S6K stimulates protein synthesis when growth factors, such as insulin, and nutrients are available (*92*, *93*). The key role of S6K downstream of insulin might stem from its signaling versatility, as it contributes to mRNA translation and ribosomal RNA synthesis thought to be limiting steps for ribosomal biogenesis, and its ability to mediate cross-talk with other key control pathways including those involved in cell cycle progression (*94*–*96*).

On the basis of the crucial role of S6K in dendritic regeneration induced by insulin, we asked whether S6K could activate mTORC2 in this process. The cross-talk between mTORC1 and mTORC2 is extensive (*97*), but the precise mechanisms underlying their interaction are unclear, particularly in the context of specific biological actions such as neuroregeneration. Our data demonstrate that S6K phosphorylates SIN1, an essential component of mTORC2 that stabilizes the interaction between mTOR and its partner rictor (*66*). Furthermore, we show that S6K is required for SIN1 phosphorylation, suggesting a cross-talk between mTORC1 and mTORC2 in adult RGCs. We show that SIN1 inhibition impaired insulin-mediated RGC dendrite regeneration after OHT induction or optic nerve axotomy, confirming a functional interaction between S6K and SIN1. The manner in which SIN1 phosphorylation affects mTORC2 activity is controversial, and mechanisms differ depending on the experimental context and the cell type. For example, a study in epithelial cancer cells found that S6K phosphorylation of SIN1 at Thr^86^ and Thr^398^ leads to SIN1 dissociation from the mTORC2 complex and inactivation (*98*), while another report in adipocytes showed that SIN1 phosphorylation at Thr^86^ enhanced mTORC2 activity (*99*). Despite similar regenerative outcome in both models, we found that insulin administration resulted in S6K phosphorylation of SIN1 at residues Thr^86^ and Thr^398^ during OHT, but only Thr^398^ in the axotomy model. Because SIN1 knockdown effectively blocked insulin-dependent dendritic growth in both injury modalities, this suggests that SIN1 phosphorylation at Thr^398^ is essential for mTORC2 activation and regenerative growth. Last, we demonstrate that SIN1 can phosphorylate Akt, which, in turn, may further activate mTORC1, resulting in a feedforward loop. Collectively, our results demonstrate a critical role of S6K/SIN1 as a link between mTORC1 and mTORC2 during insulin-mediated RGC dendrite regeneration. The precise downstream effectors that promote insulin-mediated RGC dendritic and synaptic regeneration are now unknown, but our data suggest that molecules involved in protein translation (downstream of mTORC1) and cytoskeleton dynamics (regulated by mTORC2) are likely candidates. Accumulating evidence supports the existence of local mRNA translation in dendrites and dendritic spines (*100*), which may drive protein synthesis required for insulin-induced RGC dendritic and synaptic regeneration. In neurons, mTORC2 controls actin polymerization to modify neuronal morphology and synaptic plasticity (*101*–*103*); thus, mTORC2 may promote RGC dendrite regeneration through regulation of cytoskeleton dynamics.

The ultimate goal of neuroregenerative strategies is to restore neuronal function and curtail disability. In the context of glaucoma, recovery of RGC function as well as retina-brain connectivity is paramount for vision restoration. Our data show that insulin treatment promoted neuronal survival, rescued light-evoked single-RGC Ca^2+^ transients, and improved optomotor reflex–elicited behaviors. Previous work reported a beneficial effect of insulin-like growth factor 1 on RGC axon regrowth in models of acute optic nerve injury (*104*–*106*). Although our study did not examine the effect of insulin on axon protection or regeneration, the recovery of optomotor responses, which reflect the integrity of the retinal-brain pathway, suggests that insulin exerts a neuroprotective effect on optic nerve axons. Despite continuous exposure to high IOP, insulin supported robust RGC survival, suggesting that, as a result of dendrite and synapse regeneration or concomitantly with these responses, antiapoptotic pathways were activated to enhance neuronal viability. Cumulative evidence supports a role for insulin in the regulation of glucose uptake in the brain (*107*–*109*), including the ability of insulin to induce glucose receptor translocation in neurons and astrocytes to facilitate glucose intake (*110*–*112*). Furthermore, elevated eye pressure results in increased retinal glucose levels, which may have adverse effects on RGC function and viability (*113*). Of interest, glucose administration through eye drops or subconjunctival injections promotes transient visual recovery in patients with glaucoma (*114*, *115*). On the basis of this, we cannot rule out that some of the insulin-mediated effects observed here relate to the restoration of glucose homeostasis. Furthermore, insulin receptors are expressed by other retinal cells; thus, it is possible that insulin may act on other cell types to elicit responses in RGCs. For example, Müller glia respond to insulin signaling (*116*) and have the ability to secrete neurotrophic factors, which may promote RGC survival and/or regeneration (*117*).

We report important alterations in Ca^2+^ homeostasis in RGCs subjected to OHT, notably increased Ca^2+^ decay time, which can result in cytosolic Ca^2+^ accumulation and have detrimental effects on neuronal function. Insulin shortened Ca^2+^ decay time to values similar to those found in uninjured neurons, effectively reducing cytosolic Ca^2+^ and normalizing Ca^2+^ signal dynamics. Neuronal activity and survival are intimately interconnected (*118*); thus, it is possible that, by improving synaptic connectivity, insulin enhances RGC health under stress conditions. Insulin-treated eyes displayed a significant increase in light-evoked behaviors and visual acuity, evidenced by a marked improvement of the optomotor response, supporting the conclusion that insulin restores RGC functional connectivity and promotes visual recovery. Despite robust insulin-mediated RGC neuroprotection, we observed partial recovery of visual function. We attribute this to the fact that IOP remained elevated throughout the course of the experiment, a requirement to unequivocally assess whether insulin restores visual responses independently of the confounding effect of lowering IOP. Previous work demonstrated that, despite robust neuroprotection, RGC function can be compromised by increased IOP. For example, the acetylcholinesterase inhibitor galantamine promotes robust RGC neuroprotection (~70%); however, visual evoked potentials are undetected, while IOP remains elevated (*85*). Combining galantamine with an IOP lowering drug restores visual responses in experimental glaucoma (*85*). Insulin instilled as eye drops has been shown to readily reach the retina without causing hypoglycemia, and clinical studies of insulin eye drops in healthy volunteers or patients with diabetes showed that this approach is well tolerated (*30*, *119*–*121*). In patients with glaucoma, we anticipate that insulin will be used in conjunction with the standard care to lower IOP. In summary, our study provides a compelling example of vision restoration by insulin administration offering the possibility of developing this strategy for the treatment of glaucoma and other optic neuropathies.

## MATERIALS AND METHODS

### Experimental animals

All procedures were approved by the animal protection committee of the University of Montreal Hospital Research Center and followed the Canadian Council on Animal Care guidelines. Experiments included balanced numbers of female and male adult mice (2 to 5 months) expressing the following transgenes: (i) YFP under the regulation of the Thy1 promoter (B6.Cg.Tg[Thy1-YFPH]2Jrs/J mice; the Jackson Laboratory, Bar Harbor, ME) and (ii) Ca^2+^ indicator GCaMP6f (fast kinetics) downstream of the Thy1 promoter (Thy1-GCaMP6f; the Jackson laboratory, 025393) to visualize Ca^2+^ dynamics in RGCs. For live retinal imaging, the latter line was backcrossed to an albino background (CD1, Charles River, Saint-Constant, Canada) for at least six generations. Animals were housed in 12-hour light/12-hour dark cyclic light conditions, with an average in-cage illumination level of 10 lux and fed ad libitum. Ambient temperature and humidity were maintained at 21° to 22°C and 45 to 55%, respectively. All procedures were performed under general anesthesia using 2% isoflurane (0.8 liter/min), except for the experiments involving longitudinal live recordings, which required ketamine (80 mg/kg) and xylazine (10 mg/kg).

### Magnetic microbead occlusion glaucoma model

Unilateral elevation of IOP was induced by a single injection of magnetic microbeads into the anterior chamber of the eye as we previously described in detail (*36*). Briefly, animals were anesthetized, and a drop of tropicamide was applied to the cornea to induce pupil dilation (Mydriacyl, Alcon, Mississauga, ON). A custom-made sharpened microneedle attached to a microsyringe pump (World Precision Instruments, Sarasota, FL) was loaded with 1.5 μl of a homogenized magnetic microbead solution (diameter, 4.5 μm; 2.4 × 10^6^ beads) (Dynabeads M-450 Epoxy, Thermo Fisher Scientific, Waltham, MA). Using a micromanipulator, the microneedle tip was used to puncture the cornea gently, and the microbeads were injected into the anterior chamber. A handheld magnet was used to immediately attract the magnetic microbeads to the iridocorneal angle. This procedure avoided injury to ocular structures including the lens and iris and did not interfere with the optical media clarity through the pupil. An antibiotic eye drop was applied to the operated eye (Alcon), and the animal was allowed to recover on a heating pad. IOP was measured before and after the procedure in awake animals using a calibrated TonoLab rebound tonometer (Icare, Vantaa, Finland). A drop of proparacaine hydrochloride (0.5%, Alcon) was applied to the cornea, and, holding the tonometer perpendicular to the eye surface, 10 consecutive IOP readings per eye were taken and averaged (*36*). For experiments that involved the pharmacological reduction of IOP, eye drops of the carbonic anhydrase inhibitor brinzolamide (1% w/v, Alcon) were administered twice a day (9 a.m. and 6 p.m.) for the entire duration of the experiment.

### Insulin treatment

Human recombinant insulin (Humulin-R U100, 100 U/ml, Eli Lilly) was administered as daily eye drops (5 μl drop) for the entire duration of the treatment period as specified in Results. Only the glaucomatous eye was treated with insulin or vehicle eye drops (control).

### Protein extraction and ELISA

A single insulin or saline eye drop was administered on the cornea, and, 30 min later, mice were anesthetized with isoflurane and euthanized by cervical dislocation. Retinas are dissected out and stored at −80°C until protein extraction. Lysis buffer (50 mM tris, 1 mM EDTA, 150 mM NaCl, 1% v/v NP-4O, 2 mM NaVO_3_, 5 mM NaF, and 0.25% Na deoxycholate) was added to each retina (30 to 75 μl), and tissue was grinded using a homogenizer. Samples were then centrifuged at 14,000 rpm for 5 min at 4°C, and the supernatant was collected for ELISA analysis. Insulin concentration was measured using the mouse ultrasensitive insulin ELISA kit (ALPCO Diagnostics, Salem, NH).

### RGC dendritic arbor analyses

Dendritic arbor reconstruction and measurements were performed blinded of manipulations as described (*30*, *38*, *42*). High-resolution images of YFP-labeled RGC dendrites were acquired from retinal flat-mounted preparations using a confocal microscope (Leica Microsystems Inc.) as previously described (*30*, *38*). Scans were taken at 0.5-μm intervals (1024 × 1024 pixels) with an average of three to five images per focal plane. Reconstruction of dendritic trees was carried out using the computer-aided filament tracing function of Imaris (Bitplane, South Windsor, CT). The following parameters were measured: (i) total dendritic length: the sum of the lengths of all dendrites per neuron; (ii) total dendritic field area: the area within the contour of the arbor created by drawing a line connecting the outermost tips of the distal dendrites; (iii) total number of branches: the sum of all dendritic branches per neuron; and (iv) Sholl analysis: the number of dendrites that cross concentric circles at increasing distances (10-μm interval) from the soma. RGCs located in all retinal quadrants and eccentricities were included in our analysis.

### Intraocular delivery of AAV and siRNA

The following reagents were administered intraocularly: (i) a serotype 2 AAV (AAV2) encoding RFP-labeled murine PSD95 under control of the synapsin promoter [AAV.PSD95, 3.7 × 10^12^ genome copies (GC)/ml; Vector Biolabs, Malvern, PA], (ii) control AAV2 carrying only RFP (AAV.Ctl, 1 × 10^13^ GC/ml; Vector Biolabs), (iii) siS6K (7 μg/μl, ON-TargetPlus mouse siS6K siRNA; SO-2951488G, Dharmacon, Lafayette, CO), (iv) si4EBP1 (7 μg/μl, ON-TargetPlus mouse 4EBP1 siRNA; SO-2989997G, Dharmacon), (v) siSIN1 (7 μg/μl, ON-TargetPlus mouse SIN1 siRNA; SO-3029063G, Dharmacon), or (vi) siCtl; 7 μg/μl, On-TargetPlus nontargeting siRNA; D001810-02-50, Dharmacon). A sharpened custom-made glass micropipette was inserted through the sclera into the posterior chamber to reach the vitreous cavity and deliver a total volume of 2 μl of each reagent indicated above (*38*, *63*, *64*). AAVs were injected 1 week before magnetic microbead–induced glaucoma. All injections were done into the superior-temporal quadrant of the eye and were carefully conducted to avoid injury to ocular structures or retinal detachment (*122*).

### Retinal immunohistochemistry and analysis of synaptic markers

#### Retinal cryosections

Mice were euthanized by decapitation under deep anesthesia (5% isoflurane), and the eyes were immediately collected. The cornea was carefully pierced with a 30-gauge needle, and the eye was incubated in ice-cold 4% carbodiimide (Thermo Fisher Scientific) for 30 min. Retinal cryosections (16 μm) were generated as described (*123*, *124*) and incubated with each of the following primary antibodies overnight at 4°C: VGLUT1 (1:800; Synaptic Systems, Gottingen, Germany) and PSD95 (2 μg/ml; Abcam, Cambridge, UK). Sections were washed and incubated with secondary antibodies: anti-guinea pig and anti-mouse (Alexa Fluor 594 or Alexa Fluor 488, 2 μg/ml; Molecular Probes, Eugene, OR). Three retinal cross sections per eye were analyzed in two areas (central and peripheral) for a total of six output measures per mouse. Fluorescent labeling was visualized with a Leica SP5 confocal microscope (Leica Microsystems Inc.), and 7.5-μm-thick *z*-stacks were sequentially obtained at 0.13-μm intervals (1024 × 1024 pixels) with an average of four images per focal plane. Quantitative analysis of voxels, which measured the 3D volume occupied by pre- and postsynaptic markers, was carried out using Imaris (ImarisColoc, Bitplane).

#### Flat-mounted retinas

To visualize excitatory synapses selectively on RGC dendrites, an AAV2 encoding PSD95 was administered by intravitreal injection 1 week before magnetic microbead–induced glaucoma, and eyes were collected 3 weeks later. Retinas were dissected and incubated with antibodies against NF-H (1:1000; Calbiochem, Temecula, CA, USA), YFP (0.001 μg/μl; Abcam), and RFP (0.001 μg/μl; Abcam). Only RGCs triple-labeled with YFP, PSD95 (RFP), and NF-H were selected for imaging using a Leica SP5 confocal microscope (Leica Microsystems Inc.). *Z*-stacks of each cell were sequentially acquired at 0.13-μm intervals (1024 × 1024 pixels) with an average of three images per focal plane. RGCs were classified according to their dendritic arbor morphology and stratification in the inner plexiform layer, defined as the distance between the RGC soma and the plane of dendrite ramification. Dendritic arbors and PSD95 puncta were 3D reconstructed and analyzed using the Imaris filament tracing and dot finding functions (Bitplane).

### Flow cytometry

Flow cytometry data were obtained from siRNA-mediated knockdown in conditions of injury (OHT or axotomy) and insulin treatment. A few experiments were carried out in naïve mice for siRNA validation.

#### Total protein in RGCs

Fresh retinas were cut into small pieces using scissors and incubated in a dispase (5 U/ml; STEMCELL Technologies, Vancouver, BC) and deoxyribonuclease I (2000 U/ml; Worthington Biochemical, Lakewood, NJ) solution for 20 to 25 min at 37°C in an Eppendorf thermomixer (Eppendorf, Mississauga, ON) with shaking (350 rpm). Enzymatic digestion was stopped using a blocking solution (Hanks’ balanced salt solution, 2% bovine serum albumin), and dissociated cells were washed twice with the same solution. The LIVE/DEAD Fixable Aqua Dead Cell Stain Kit (Thermo Fisher Scientific) was used to determine the viability of cells before the fixation and permeabilization required for intracellular antibody staining. Cells were washed in fluorescence-activated cell sorting (FACS) buffer (phosphate-buffered saline with 1% fetal bovine serum and 0.1% sodium azide), fixed, and permeabilized in a paraformaldehyde (PFA)/saponin buffer prewarmed at 37°C. Cells were incubated in the following primary antibodies: RBPMS (0.25 μg/ml; PhosphoSolutions, Aurora, CO), S6K (0.3 μg/μl; Proteintech, Rosemont, IL), 4EBP1 (0.02 μg/μl; Invitrogen, Carlsbad, CA), or SIN1 (2 μg/μl; Abcam) for 60 min at 4°C, followed by incubation in secondary antibodies (Alexa Fluor 647 or Alexa Fluor 488, 0.4 μg/μl; Invitrogen), and washed twice before flow cytometry. Data were acquired with a BD LSRII flow cytometer using Diva software (BD Biosciences, Mississauga, ON) and analyzed using the FlowJo software (FlowJo, Ashland, OR). Control cells were incubated with only secondary antibodies or with the primary antibody and a secondary antibody from a different species to rule out nonspecific binding.

#### Phosphorylated protein expression in RGCs

Dissociated retinal cells were fixed in PFA (1.5%) for 10 min at 37°C, permeabilized in Phosflow Perm Buffer III (BD Biosciences), and incubated with antibodies against phospho-SIN1 at Thr^86^ (pSIN1-Thr^86^, 0.2 μg/μl; Cell Signaling Technology, Danvers, MA), phospho-SIN1 at Thr^398^ (pSIN1-Thr^398^, 1 μg/μl; EMD Millipore, Temecula, CA) or phospho-Akt at Ser^473^ (pAkt-Ser^473^, 5:50; BD Biosciences), phospho-Akt at Thr^308^ (pAkt-Thr^308^, 1:50; Cell Signaling Technology), and RBPMS (0.4 μg/μl; PhosphoSolutions) for 60 min at 4°C in the dark. Cells were washed twice in FACS buffer and incubated with secondary antibodies for 30 min, followed by two additional washes. Data were acquired using the BD LSRII flow cytometer (BD Biosciences) and analyzed with FlowJo software (FlowJo LLC, BD Life Sciences, Ashland, OR).

### Optic nerve axotomy

Axonal injury was induced by complete transection (axotomy) of the optic nerve to trigger rapid and stereotypical RGC loss as described (*30*, *38*, *63*). Briefly, an incision in the skin over the superior orbital rim was made to gain access to the back of the eye. The dural sheath was longitudinally opened to expose the optic nerve, which was then cleanly transected at 0.5 to 1 mm from the back of the globe. Care was taken not to damage the central retinal artery and fundus examination was routinely performed before and after the procedure to verify the integrity of the retinal circulation. Animals showing compromised blood supply were excluded from the study.

### Two-photon microscopy live imaging of Ca^2+^ dynamics

Live imaging of light-evoked Ca^2+^ transients in single RGCs was performed by TPLSM as described (*43*, *55*, *71*). Mice were anesthetized and placed on a customized platform with controlled temperature (37°C) and air ventilation. The eyelids were opened, and a surgical 6.0 suture attached to the surrounding ocular muscle was used to expose the sclera in the superior temporal retina. The conjunctiva was gently teased away to place a 5-mm-diameter coverslip directly against the sclera (Harvard apparatus, Holliston, MA), which served as a flat plane to place the microscope objective. Live Ca^2+^ imaging was recorded from single RGCs in Thy1-GCaMP6f transgenic mice to obtain baseline recordings. Mice were randomly assigned to the following experimental groups: sham, OHT + insulin, or OHT + vehicle. The animals were subjected to a second TPLSM recording 3 or 4 weeks after glaucoma induction. GCaMP6f-positive RGC soma were scanned at 12 Hz using a 20× water-immersion objective in multiphoton microscope (Zeiss). Excitation was set at 920 nm, using a Ti:sapphire laser (Chameleon Ultra, Coherent). For light-triggered visual stimulation, a flash stimulus (102 cd/m^2^, 6 ms) was generated with a PowerLab unit (ADInstruments, Colorado Springs, CO). The stimulus was presented using a white light-emitting diode located 5 mm away from the corneal apex and centered relative to the pupil as previously described (*43*, *55*). Stimulus onset and TPLSM imaging recordings were synchronized offline by identifying the frame where the light flash was registered. Light-evoked Ca^2+^ responses were measured by averaging the fluorescence intensity of all pixels within the region of interest after background subtraction using ImageJ. The following Ca^2+^ transient parameters were calculated: (i) rise rate, defined as the slope of the rising phase (Δ*F*/*F*_0_/ΔTime) determined by best fit linear regression model due to fast Ca^2+^ kinetics during this phase (*125*); (ii) decay time, defined as the exponential decay time constant (τ) of Ca^2+^ signals obtained from single exponential curve fitting (*73*); and (iii) amplitude, defined as Δ*F*/*F*0 peak, where *F*0 is the baseline fluorescence signal averaged over a 2-s period before the beginning of the light stimulus. A freely available custom R routine was used for data analyses (www.r-project.org).

### RGC survival quantification

Animals were transcardially perfused with 4% PFA, and retinas were dissected out and incubated for 3 days with an antibody against RBPMS (0.25 μg/μl; PhosphoSolutions) followed by an Alexa Fluor 647–conjugated secondary antibody (4 μg/μl; Invitrogen) as described (*42*, *43*, *63*). Retinas were washed and mounted using an antifade reagent (SlowFade, Molecular Probes). Images were obtained using an Axio Imager M2 optical sectioning microscope (20× objective, Zeiss) equipped with an automated stage for *x*-, *y*-, and *z*-axis movement, a color camera (Axiocam 509 mono, Zeiss), and image analysis software (Zen, Zeiss). We used an unbiased stereological approach based on systematic uniform random sampling from 3D dissectors (stacks) across the entire retina, and images were acquired using identical exposure time and gain settings for all experimental and control groups. RGC density was quantified using a custom-made quadrant dissector in Fiji/ImageJ.

### Optomotor response assays

Visual acuity was evaluated by measuring the optomotor reflex using the OptoMotry virtual reality system (Cerebral Mechanics Inc., Medicine Hat, AB, Canada), which allows the quantification of visuomotor behaviors in response to visual stimuli (*55*). The animals were placed on an elevated platform in the center of a testing arena with walls composed of computer monitors displaying a rotating vertical black and white sine-wave grating pattern. The staircase method was used to determine the spatial frequency. Both contrast (100%) and rotation speed (12/s) were kept constant. Cameras were used to record the animals, and an observer masked to treatment monitored their tracking behavior. A behavioral response was considered positive when the motor response (head movement) was concordant with the direction of the visual stimulus (moving bars). Individual scores from each mouse were collected before and after each treatment.

### Statistical analyses

Data analysis was always carried out blinded by third-party concealment of treatment using uniquely coded samples. For each dataset, “*N*” represents the number of mice used and “*n*” represents the number of cells analyzed, and these are indicated in the figure legends. All cohorts were evaluated with normality (Shapiro-Wilk test) and variance (*F* test) tests. Statistical analyses were performed using GraphPad Instat software (GraphPad Software Inc., San Diego, CA) by a one-way analysis of variance (ANOVA) followed by a Bonferroni or Tukey post hoc tests or by a Student’s *t* test as indicated in the legends. A *P* value of ≤0.05 was considered significant.
