# Supplementary material for: Insulin restores retinal ganglion cell functional connectivity and promotes visual recovery in glaucoma
Source: Sci Adv. 2024 Aug 7;10(32):eadl5722. doi: 10.1126/sciadv.adl5722 (PMC11305393; doi:10.1126/sciadv.adl5722)
Supplement: Supplementary file 1 — Figs. S1 to S6 [file sciadv.adl5722_sm.pdf]

Science Advances
AAAS

Supplementary Materials for

Insulin restores retinal ganglion cell functional connectivity and promotes visual recovery in glaucoma

Sana El Hajji *et al.*

Corresponding author: Adriana Di Polo, adriana.di.polo@umontreal.ca

Sci. Adv. **10**, eadl5722 (2024)
DOI: 10.1126/sciadv.adl5722

This PDF file includes:

Figs. S1 to S6

Fig. S1

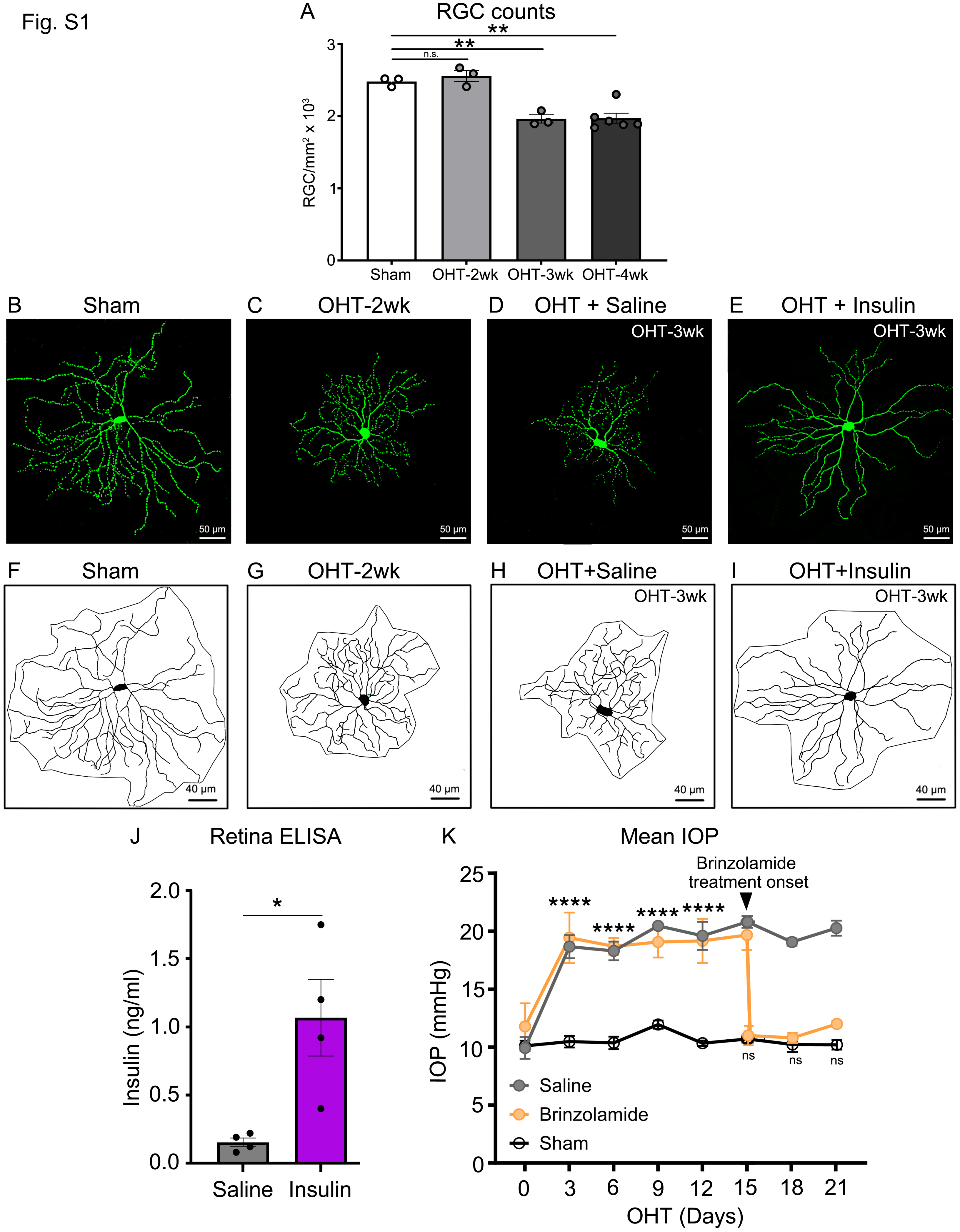
A
RGC counts
RGC/mm² x 10³
n.s.
**
**
Sham
OHT-2wk
OHT-3wk
OHT-4wk
B
Sham
C
OHT-2wk
D
OHT + Saline
OHT-3wk
E
OHT + Insulin
OHT-3wk
50 µm
F
Sham
G
OHT-2wk
H
OHT+Saline
OHT-3wk
I
OHT+Insulin
OHT-3wk
40 µm
J
Retina ELISA
Insulin (ng/ml)
*
Saline
Insulin
K
Mean IOP
Brinzolamide treatment onset
IOP (mmHg)
****
ns
Saline
Brinzolamide
Sham
OHT (Days)

Fig. S2

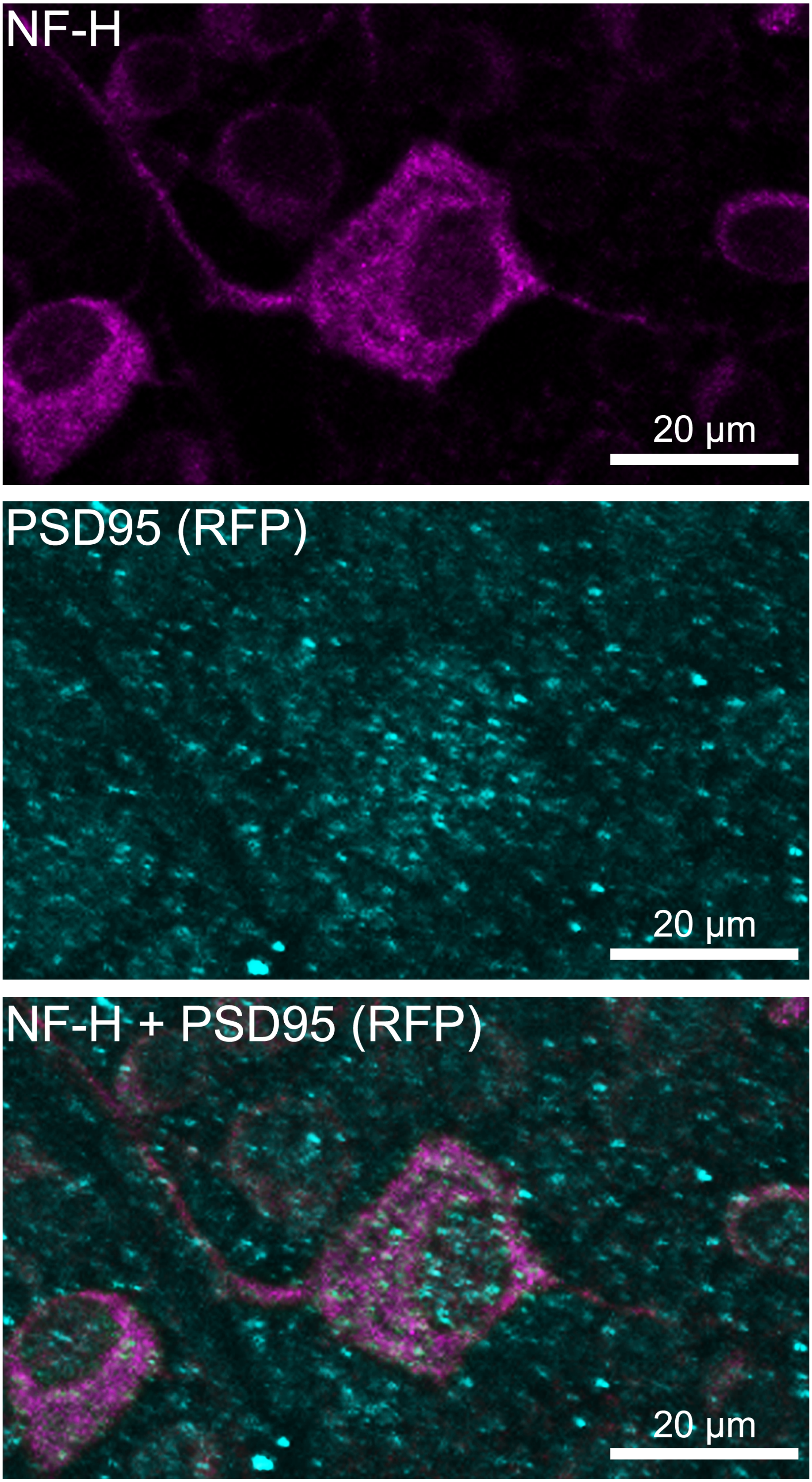

Fig. S3

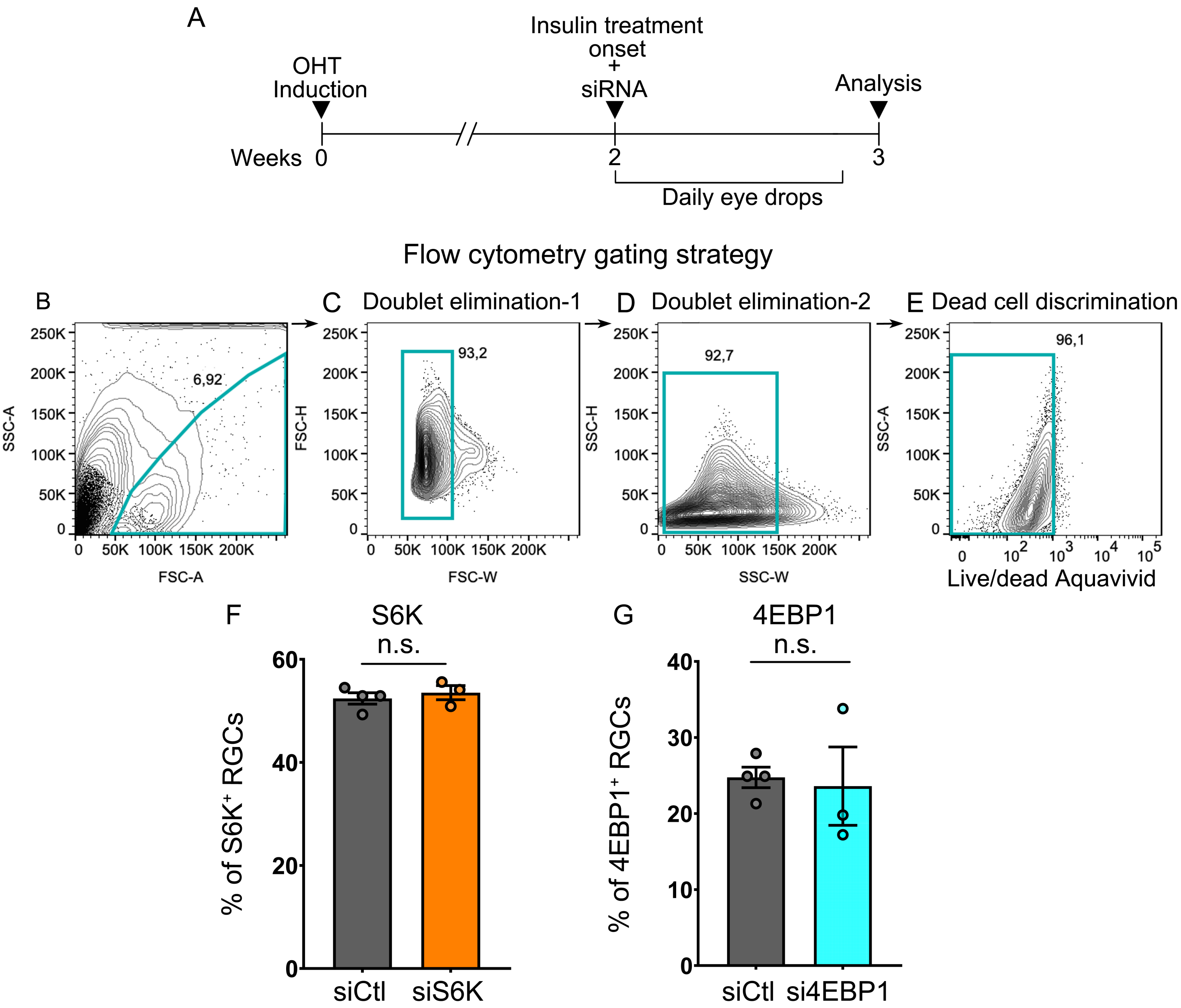
A
OHT Induction
Insulin treatment onset + siRNA
Analysis
Weeks 0
2
3
Daily eye drops
Flow cytometry gating strategy
B
6,92
SSC-A
FSC-A
C Doublet elimination-1
93,2
FSC-H
FSC-W
D Doublet elimination-2
92,7
SSC-H
SSC-W
E Dead cell discrimination
96,1
SSC-A
Live/dead Aquavivid
F
S6K
n.s.
% of S6K⁺ RGCs
siCtl
siS6K
G
4EBP1
n.s.
% of 4EBP1⁺ RGCs
siCtl
si4EBP1

Fig. S4

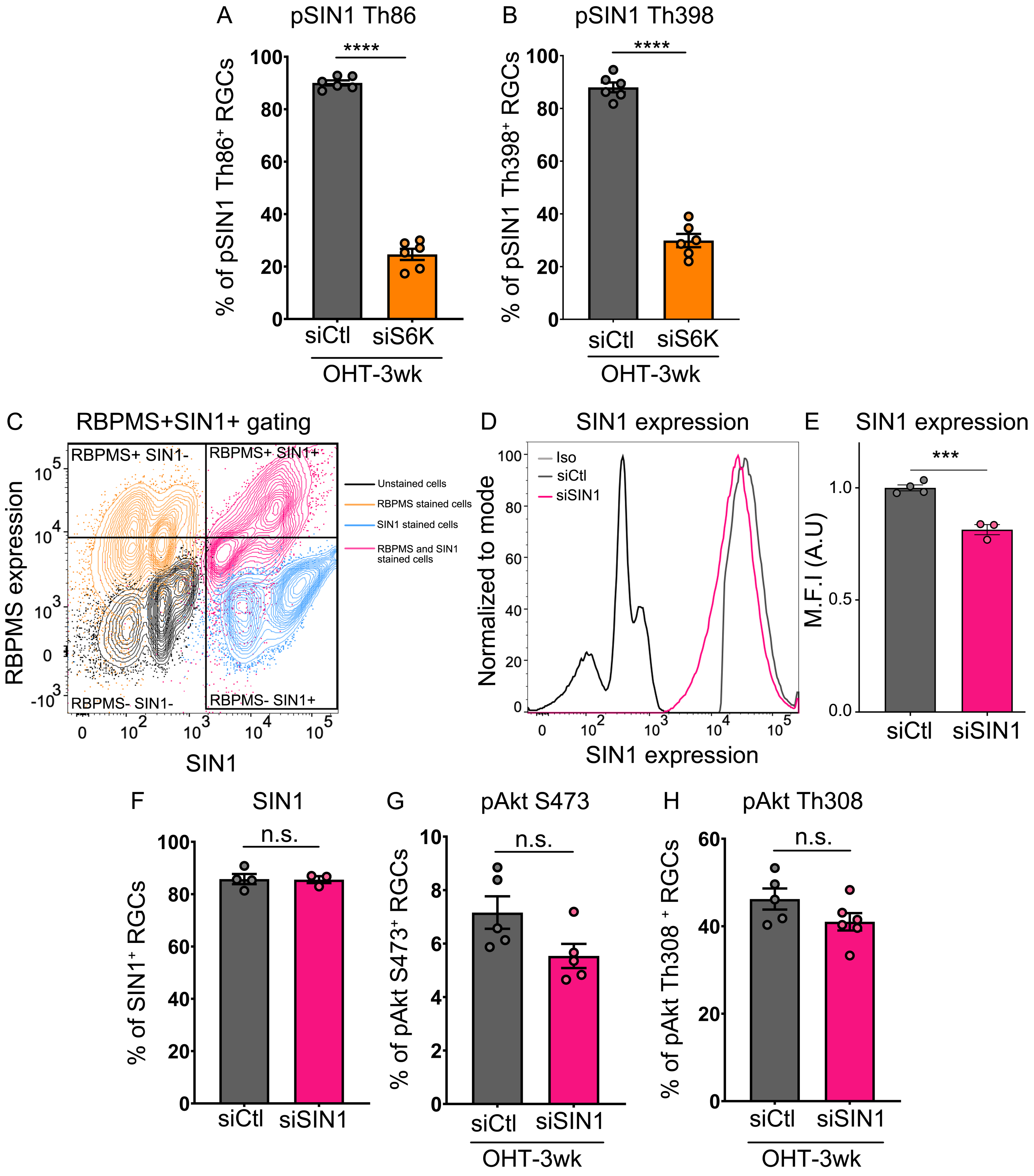

A
pSIN1 Th86
% of pSIN1 Th86+ RGCs
****
siCtl
siS6K
OHT-3wk
B
pSIN1 Th398
% of pSIN1 Th398+ RGCs
****
siCtl
siS6K
OHT-3wk
C
RBPMS+SIN1+ gating
RBPMS+ SIN1-
RBPMS+ SIN1+
RBPMS- SIN1-
RBPMS- SIN1+
RBPMS expression
SIN1
Unstained cells
RBPMS stained cells
SIN1 stained cells
RBPMS and SIN1 stained cells
D
SIN1 expression
Iso
siCtl
siSIN1
Normalized to mode
SIN1 expression
E
SIN1 expression
***
M.F.I (A.U)
siCtl
siSIN1
F
SIN1
n.s.
% of SIN1+ RGCs
siCtl
siSIN1
G
pAkt S473
n.s.
% of pAkt S473+ RGCs
siCtl
siSIN1
OHT-3wk
H
pAkt Th308
n.s.
% of pAkt Th308 + RGCs
siCtl
siSIN1
OHT-3wk

Fig. S5

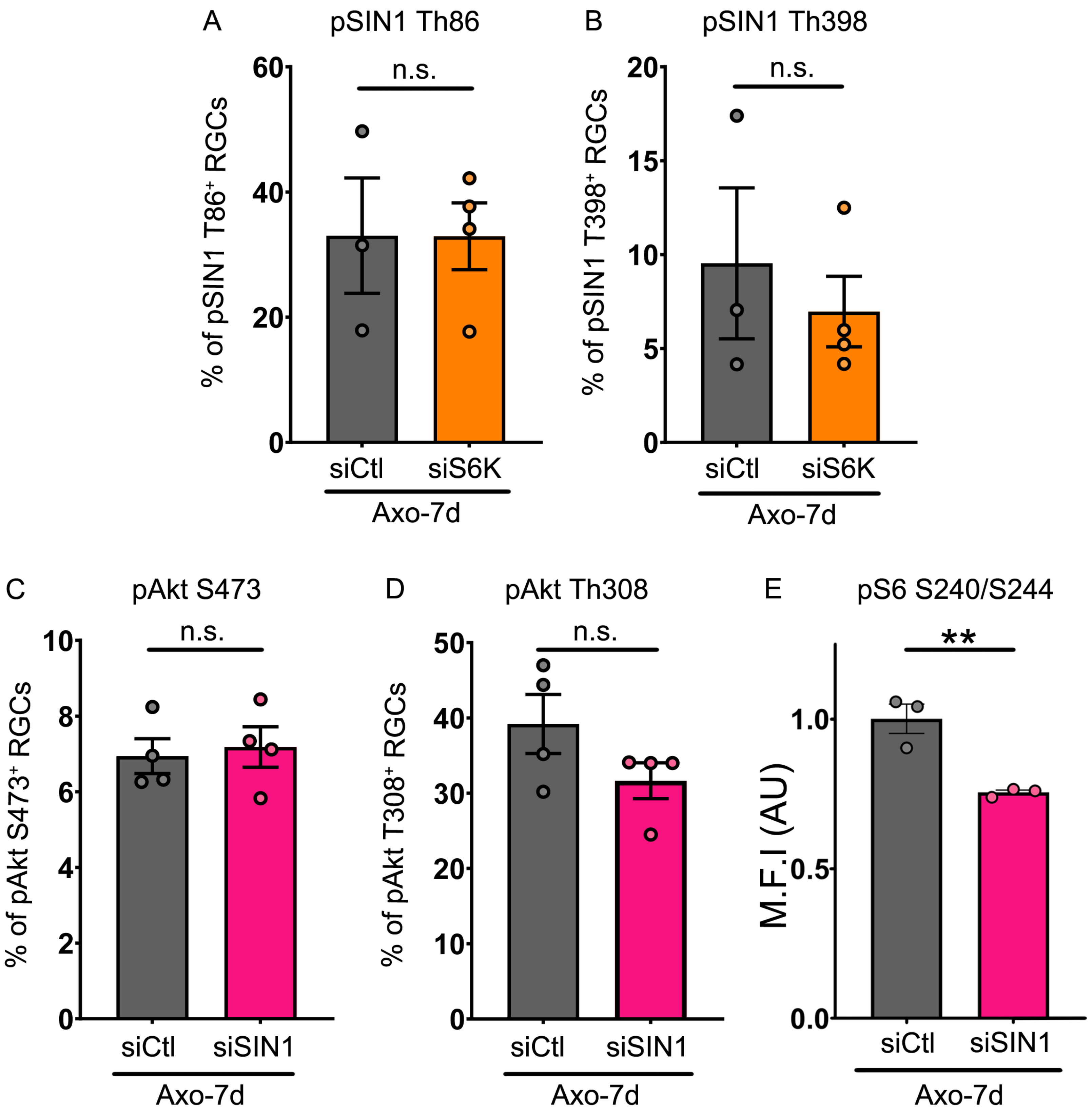

A
pSIN1 Th86
60
40
20
0
n.s.
% of pSIN1 T86⁺ RGCs
siCtl
siS6K
Axo-7d
B
pSIN1 Th398
20
15
10
5
0
n.s.
% of pSIN1 T398⁺ RGCs
siCtl
siS6K
Axo-7d
C
pAkt S473
10
8
6
4
2
0
n.s.
% of pAkt S473⁺ RGCs
siCtl
siSIN1
Axo-7d
D
pAkt Th308
50
40
30
20
10
0
n.s.
% of pAkt T308⁺ RGCs
siCtl
siSIN1
Axo-7d
E
pS6 S240/S244
1.0
0.5
0.0
**
M.F.I (AU)
siCtl
siSIN1
Axo-7d

Fig. S6

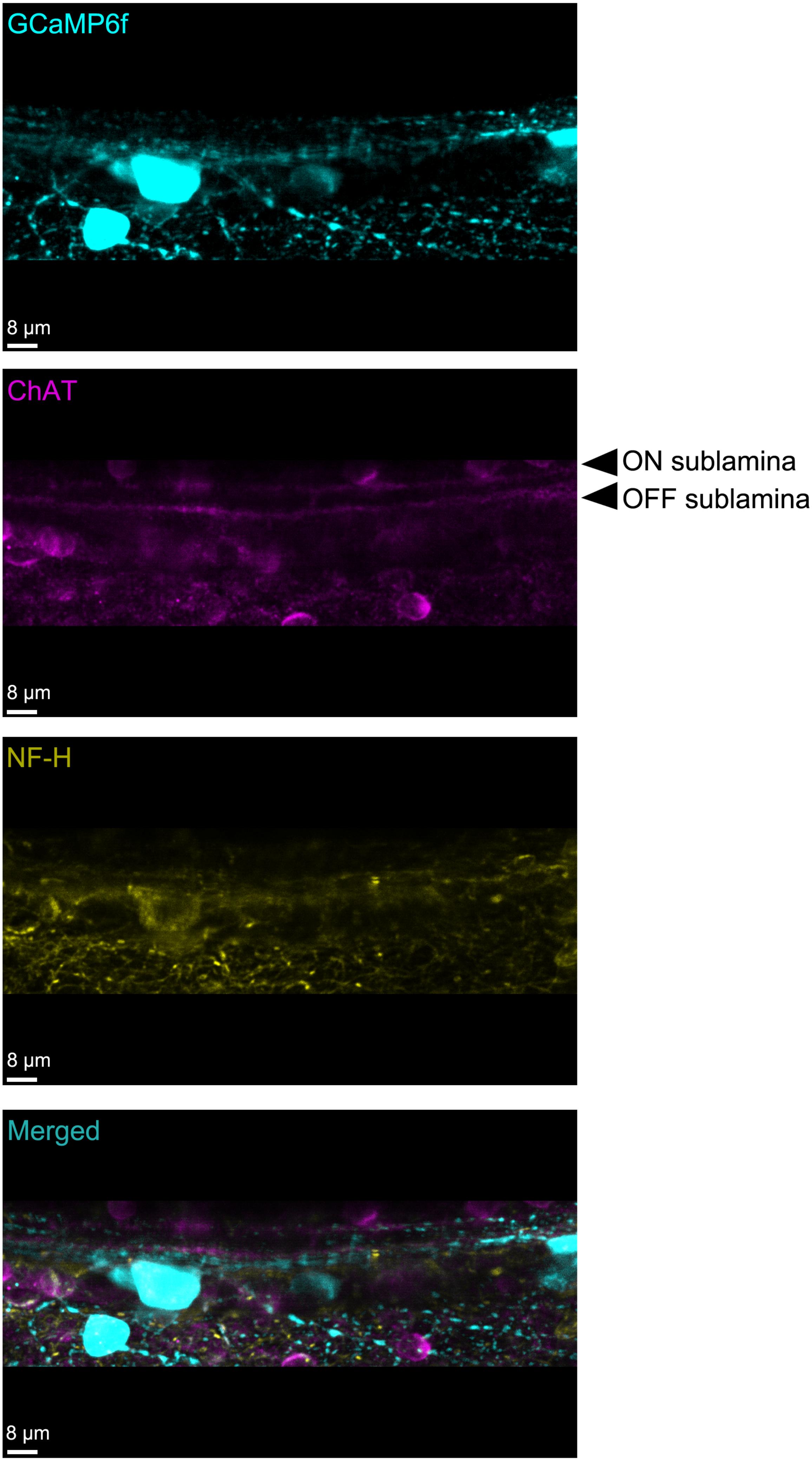
GCaMP6f
8 μm
ChAT
ON sublamina
OFF sublamina
8 μm
NF-H
8 μm
Merged
8 μm

SUPPLEMENTARY FIGURE LEGENDS

Supplementary Figure 1. Insulin treatment regimen and the effect of brinzolamide on intraocular pressure regulation.

A Quantitative analysis of RBPMS$^+$ RGCs demonstrates that there is no significant cell death at two weeks after OHT induction relative to sham-operated controls (N=3 mice/group, ANOVA, n.s.=not significant). At three and four weeks of OHT there was a significant loss of RGCs compared to sham controls (N=3-4 mice/group, ANOVA, **p<0.001). **B-E** Representative confocal images of YFP-filled RGCs show dendritic arbours from sham-operated controls (B), glaucomatous retinas at two weeks of OHT (C), and glaucomatous retinas (OHT-3wk) treated with saline (D) or insulin (E) eyedrops. **F-I** Images in B-E following three-dimensional reconstruction are shown as skeletonized dendritic arbours and the dendritic area used for analysis is outlined. **J** Quantification of insulin levels by ELISA from retinal samples collected 30 min after a single insulin or saline eyedrop administration (N=4 mice/group, Student's t-test, *P<0.01). **K** Brinzolamide daily eyedrops effectively reduced intraocular pressure (IOP) after magnetic microbead intracameral injection to levels similar to sham uninjured controls (N=5-6 mice/group, ANOVA, ****p<0.0001) (Table 3).

Supplementary Figure 2. Identification of αRGCs for synaptic puncta analysis.

For analysis of AAV-mediated PSD95$^+$ synaptic puncta, we focused on αRGCs, identified by their NF-H expression in combination with red fluorescent protein (RFP), the tag to visualize AAV-encoded PSD95.

Supplementary Figure 3. siRNA administration timeline and flow cytometry gating strategy for RGC-specific protein expression quantification.

A Timeline of regimen used to test the effect of combined siRNA and insulin administration. siRNA intraocular injection was performed at two weeks of OHT induction concomitant with the onset of insulin treatment and eyes were analyzed one week later (3 weeks after OHT). **B-E** Flow cytometry contour plots showing the gating strategy to select live cells after retinal dissociation. The first population was selected based on the size and complexity of the cells (B) and doublet cells are then eliminated (C, D). Dead cells are eliminated using AquaVivid staining test (E). **F, G** siS6K or si4EBP1 administration did not change the percentage of RGCs expressing S6K or 4EBP1, respectively, relative to the total number population (RBPMS$^+$ cells) compared to siCtl-treated retinas (N=3-4 mice/group, Student's t-test, n.s.= not significant)..

Supplementary Figure 4. Flow cytometry analysis of SIN1 expression in RGCs and the effect of SIN1 knockdown

A, B siS6K administration reduced the number of RGCs expressing pSIN1 Th86$^+$ and pSIN1Th398$^+$ in the total neuronal population (RBPMS$^+$ cells) compared to siCtl-treated retinas in OHT conditions. **C** Flow cytometry contour plot showing the gating strategy to select RBPMS$^+$SIN1$^+$ RGCs. The gates were set based on the fluorescence minus one (FMO) controls, cells that are RBPMS$^-$SIN1$^-$, RBPMS$^+$SIN1$^-$, and RBPMS$^-$SIN1$^+$ (N=6 mice/group, Student's t-test, ****$p<0.0001$). **D** Flow cytometry histogram showing that SIN1 knockdown reduces RGC-specific SIN1 expression relative to siCtl (N=3-4 mice/group, Student's t-test, ***$p<0.001$). **E** Quantitative analysis of flow cytometry data confirmed the efficacy of SIN1 knockdown with siSIN1. **F** siSIN1 administration did not change the percentage of RGCs expressing SIN1 in the total RGC population (RBPMS$^+$ cells) compared to siCtl-treated retinas (N=3-4 mice/group, Student's t-test, n.s.= not significant). An isotype (Iso) non-targeting antibody is included as control. **G, H** siSIN1 administration did not change the percentage

of RGCs expressing pAktS473^{+} or pAkt Th308^{+} SIN1 in the total RGC population (RBPMS^{+} cells) compared to siCtl-treated retinas in OHT (N=5-6 mice/group, Student's t-test, n.s.= not significant).

Supplementary Figure 5. Analysis of pSIN1 and pAkt levels in axotomized RGCs after siS6K or siSIN1 treatment

A, B siS6K administration reduced SIN1 phosphorylation at Th398, but not at Th86 (Fig. 6C, D), without reducing the percentage of pSIN1Th86^{+} and pSIN1Th398^{+} RGCs in the total population (RBPMS^{+} cells) compared to siCtl-treated retinas after axotomy (N=3-4 mice/group, Student's t-test, n.s.= not significant). **C, D** siSIN1 administration did not change the percentage of pAkt S473^{+} and pAkt Th308^{+} cells in the total population (RBPMS^{+} cells) compared to siCtl-treated retinas after axotomy (N=4 mice/group, Student's t-test, n.s.= not significant). **E** siSIN1 administration significantly reduced the phosphorylation of the ribosomal protein S6, a reliable downstream marker of mTORC1 function (N=3 mice/group, Student's t-test, $^{**}p<0.01$.)

Supplementary Figure 6. Analysis of GCaMP6 retinas to confirm the identity of ON αRGCs.

Following the recording of light-evoked Ca^{2+} signals using two-photon laser scanning microscopy, the identity of ON αRGCs was confirmed by *post hoc* immunohistochemical analysis of NF-H expression as well as dendritic stratification in the ON sublamina. ChAT labeling serves as reference for ON and OFF sublamina (arrowheads) and confirms αON-S RGC dendritic stratification in the proximal ON sublamina.
